# Neural correlates of up-regulating positive emotions in fMRI and their link to affect in daily life

**DOI:** 10.1093/scan/nsz079

**Published:** 2019-10-31

**Authors:** Johanna M Grosse Rueschkamp, Annette Brose, Arno Villringer, Michael Gaebler

**Affiliations:** 1 Humboldt-Universität zu Berlin, Institute of Psychology, 10099 Berlin, Germany; 2 MindBrainBody Institute at the Berlin School of Mind and Brain, Humboldt-Universität zu Berlin, 10099 Berlin, Germany; 3 Stroke Center Berlin and NeuroCure Cluster of Excellence, Charité—Universitätsmedizin Berlin, 10117 Berlin, Germany; 4 Department of Neurology, Max Planck Institute for Human Cognitive and Brain Sciences, 04103 Leipzig, Germany

**Keywords:** positive emotions, experience sampling, ventral striatum, affect, up-regulation

## Abstract

Emotion regulation is typically used to down-regulate negative or up-regulate positive emotions. While there is considerable evidence for the neural correlates of the former, less is known about the neural correlates of the latter—and how they are associated with emotion regulation and affect in daily life. Functional magnetic resonance imaging (fMRI) data were acquired from 63 healthy young participants (22 ± 1.6 years, 30 female), while they up-regulated their emotions to positive and neutral images or passively watched them. The same participants’ daily affect and emotion regulation behavior was measured using experience sampling over 10 days. Focusing on the ventral striatum (VS), previously associated with positive affective processing, we found increased activation during the up-regulation to both positive and neutral images. VS activation for the former positively correlated with between- and within-person differences in self-reported affective valence during fMRI but was not significantly associated with up-regulation in daily life. However, participants with lower daily affect showed a stronger association between changes in affect and activation in emotion-related (medial frontal and subcortical) regions—including the VS. These results support the involvement of the VS in up-regulating positive emotions and suggest a neurobehavioral link between emotion-related brain activation and daily affect.

Our emotional experiences are characterized by ups and downs. While these changes depend on situations we encounter, we also influence how we feel by deliberately up- or down-regulating our emotions. There are different motivations to do so, but, in general, people are pro-hedonically motivated, that is, they want to maintain or increase their positive and decrease their negative emotions (Riediger *et al*., 2009). Previous neuroimaging studies have mainly focused on the down-regulation of negative emotions and identified brain regions or networks supporting this type of regulation: most often, ‘cognitive control’ regions in prefrontal and parietal cortices have been shown to modulate subcortical regions involved in emotional responding (e.g. amygdala; Buhle *et al*., 2014). However, people can also pursue pro-hedonic goals by enhancing positive emotions. While behavioral studies in the laboratory (Giuliani *et al*., 2008) and in daily life (Jose *et al*., 2012) found that up-regulating positive emotions can enhance momentary levels of affect, less is known about the brain regions underlying this form of emotion regulation and the heightened experience of affect.

One of the brain structures suggested to be involved in—particularly positive—affective processing is the ventral striatum (VS). The VS has been implicated specifically in reward-related behavior (Schultz *et al*., 1997; Kringelbach and Berridge, 2009) and more generally in positive emotional responding, for example, to pleasant music (Blood and Zatorre, 2001), smiling faces (Vrticka *et al*., 2011) or positive images (Sabatinelli *et al*., 2007). Furthermore, VS activity can be modulated through emotion regulation, for example by cognitive reappraisal, which can increase positive emotions in negative contexts (Doré *et al*., 2017). Such regulatory effects are usually ascribed to cognitive control processes in prefrontal and parietal regions (such as lateral and medial prefrontal as well as lateral parietal cortices), which in turn modulate activity in subcortical affect processing regions, such as the VS and the amygdala (Wager *et al*., 2008; Ochsner *et al*., 2012). Thus, to the extent that the up-regulation of positive emotions successfully enhances positive affective experiences, it should modulate activity in the VS.

Indeed, the few existing functional magnetic resonance imaging (fMRI) studies that examined the up-regulation of positive emotions reported increased activation in the VS—along with activation in medial and lateral prefrontal areas (similar to the down-regulation of negative emotion), the temporal lobe and the anterior cingulate (Kim and Hamann, 2007; Vrticka *et al*., 2011; Greening *et al*., 2014; Moutsiana *et al*., 2014; Li *et al*., 2018). In one of these studies, increased VS activity was related to behavioral measures of regulation success, that is, higher positive affect during up-regulating compared to just watching positive stimuli (Greening *et al*., 2014). However, several aspects of the role of the VS during the up-regulation of positive emotions remain unknown.

First, previous studies that found increased activation in the VS during the up-regulation of positive emotions used a condition of ‘naturally’ viewing positive stimuli as a baseline. This way, one contrasts regulatory processes on the one hand and passive states on the other. To disentangle neural responses of the up-regulation of positive emotions from other regulatory processes, an ‘active’ control condition is needed. The up-regulation to neutral stimuli is such an active control condition, used to induce minimal affect (Gasper, 2018). Based on reports that the VS supports the heightened experience of positive affect during emotion regulation (e.g. Doré *et al*., 2017), we hypothesized stronger VS activation during the up-regulation to positive than to neutral stimuli, as the latter should not lead to changes in momentary affect.

Second, while activation in the VS has been related to between-person differences in the ability to up-regulate positive emotions (i.e. individuals with more activation have higher positive affect; Greening *et al*., 2014), it is important to also consider variability ‘within’ individuals. A relation between VS activity and within-person changes in affect would indicate that, in addition to being persistently activated across contexts, the VS also reflects more subtle moment-to-moment changes in affect during the up-regulation of positive emotions. Such dynamic changes in affective states have also been associated with reward-related learning processes in the VS (Rutledge *et al*., 2014; Eldar *et al*., 2016). For example, exaggerated reward expectations during heightened positive affective states lead to decreases in positive affect. Lower affective states then facilitate increases in positive affective experiences through adjusted reward expectations (Eldar and Niv, 2015; Eldar *et al*., 2016). Combined with the relation between VS activity and differences in affect during the up-regulation of positive emotions (e.g. Greening *et al*., 2014), we hypothesized that activation in the VS also reflects within-person changes in affect during the up-regulation of positive emotions. Understanding the neural responses that support these brief changes in affect is particularly relevant considering the unpredictability of everyday life situations. Ever-changing contexts and an individual’s interaction with them naturally result in varying regulatory efforts and varying affective states.

To investigate an association between brain activation and moment-to-moment changes in affect—and to determine its generalizability (Araújo *et al*., 2007)—it is beneficial to test individuals in the lab as well as their ‘natural habitat’. For example, Heller *et al.* (2015) investigated the link between reward and positive emotional states (cf. Eldar *et al*., 2016) using a (rewarded) game and affect ratings in both the fMRI and in daily life. Their finding of a positive association between sustained reward-related VS activity and sustained positive affect in daily life suggests common pathways for affect-related brain activation (as measured in the lab) and the dynamics of emotional experience in daily life. Combining fMRI and daily life measures thus allows a better understanding of how neuroaffective processes relate to the experience of positive affect in daily life; thereby assessing the real-world relevance of lab-based neuroscientific findings. Assuming a similarity of behavior in- and outside the laboratory, we expected that increased VS activity during emotion regulation in the fMRI also relates to changes in momentary affect when up-regulating positive emotions in daily life.

Taken together, in the present study, we investigated the neurobehavioral associations of the up-regulation of positive emotions during fMRI and in daily life. First, a standard emotion regulation paradigm was used to measure neural and behavioral responses while participants were instructed to up-regulate their affect to positive and neutral images during fMRI—compared to passively watching them. Given its above-mentioned involvement in positive affective processing, the present study focused on the role of the VS for the heightened experience of affect during the up-regulation of positive emotions. We tested three hypotheses: (i) the VS is recruited more strongly when up-regulating to positive images compared to just watching them and to up-regulating to neutral images; (ii) higher VS activation is related to higher between-person levels of affect during up-regulation; and (iii) higher VS activation is related to higher within-person changes in affect during up-regulation (i.e. on a trial-by-trial basis).

Second, participants completed an additional 10 days of smartphone-based experience sampling in their daily lives, during which they reported their momentary affect and degree of regulating positive emotions. Given the small empirical basis with a similar approach, we explored whether stronger activation in the VS during instructed up-regulation in the laboratory is related to higher changes in momentary affect when up-regulating in daily life.

**Fig. 1:**
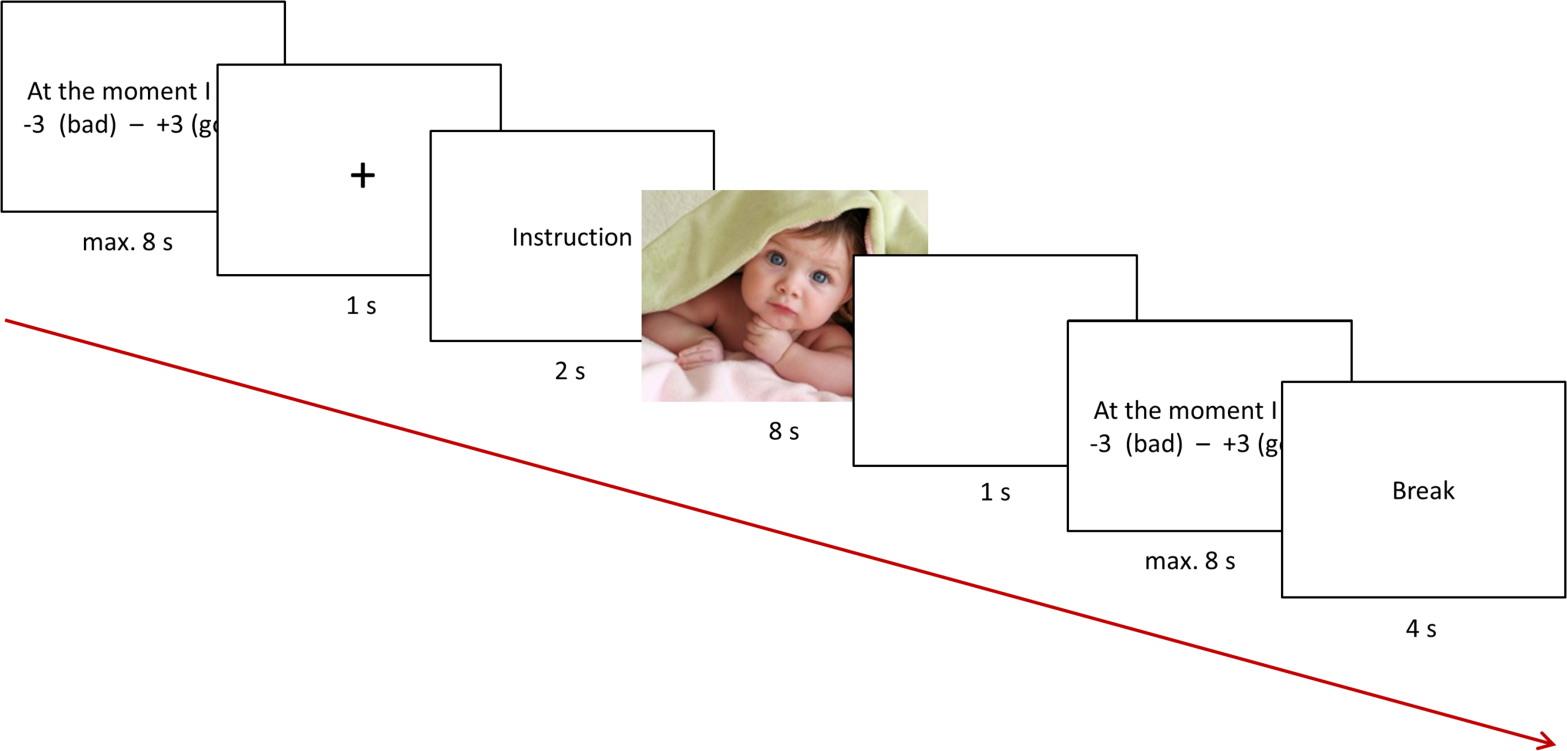
Schematic of one trial in the emotion regulation task: (i) pre-image affect rating (trial continued when answer was given), (ii) fixation cross, (iii) instruction cue word (‘Enhance’ or ‘Watch’), (iv) inter-stimulus interval, (v) post-image affect rating (trial continued when answer was given) and (vi) short break.

## Materials and methods

### Participants

A total of 77 healthy participants between 18 and 25 years (*M* = 22, *SD* = 1.6, 39 women) were recruited through mailing lists and online ads. Exclusion criteria were current psychiatric or neurological disorders, an above-normal body mass index (18.5–25 kg/m^2^) and standard MRI contraindications (e.g. metallic implants). Data from two participants were excluded due to technical issues (wrong MRI sequence parameters and crashing task presentation) and two participants decided to terminate their participation. After a more detailed screening during the testing session, an additional 10 participants were excluded because of a history of neurological or psychiatric diagnoses. Hence, 63 participants (*M* = 22, *SD* = 1.6, 30 women) entered the analyses.

### Procedure

The experiment comprised two phases, an fMRI and an experience sampling method (ESM) phase, the order of which was counterbalanced across participants (49% fMRI first). During the ESM introductory session, participants received smartphones and completed trait questionnaires (not relevant for the current research question; Supplement 1.1). During fMRI, an emotion regulation task and a reward-learning task (the results of which will be presented elsewhere) were performed. Both tasks were practiced beforehand outside the scanner. Participant reimbursement ranged from 44.50 to 90 euros, depending on the performance in the reward-learning task and the number of completed ESM measurement occasions. The study was approved by the ethics committee of the medical faculty at the University of Leipzig.

### Emotion regulation task in the MRI

A total of 40 positive (Pos; valence: *M* = 7.09, *SD* = 0.34; arousal: *M* = 4.59, *SD* = 0.72) and 40 neutral images (Neu; valence: *M* = 5.29, *SD* = 0.17; arousal: *M =* 3.15, *SD* = 0.40) were chosen as stimuli from the Emotional Picture Set (EmoPicS; Wessa *et al*., 2010) based on the normative ratings (9-point Self-Assessment Manikins: 1 = sad/calm, 9 = happy/excited) and matched between conditions for number of persons depicted, social interactions, close-up images and eye contact. Participants were instructed to either up-regulate their emotions (‘deliberately intensify the emotions you are experiencing’; Up) or to passively watch (‘experience the emotions naturally as they come and go’; Watch) indicated by the cue words ‘Enhance’ or ‘Watch’ (for exact wording in German, see Supplement 1.2). No specific emotion regulation strategy was instructed, as we aimed to maximize the comparability with the assessment in daily life, where people report using several emotion regulation strategies (Heiy and Cheavens, 2014). Each of the four experimental conditions (PosUp, PosWatch, NeuUp, NeuWatch) had 20 trials, split into two runs of 40 trials each. For each participant, images were randomly assigned to the four conditions and the trial order was pseudo-randomized with the constraint of maximally three consecutive trials from the same condition. Before and after each image, participants rated their momentary affective valence (‘At the moment I feel …’) on a scale from −3 (‘bad’) to +3 (‘good’; see Figure 1) by using an MRI-compatible box with three buttons. The rating always started at ±0. The left button de- and the right button increased the rating, while the middle button confirmed it. The currently chosen option was visually highlighted. After each fMRI session, participants were asked how much they engaged in up-regulation during the task and how strongly they used each of four different emotion regulation strategies (for details, see Supplementary Table S1).

### Experience sampling in daily life

During the 10-day ESM phase (two periods of 5 days, separated by a 2-day break), participants answered questions on a smartphone (Huawei Ascend G330), which beeped six times per day at pseudo-random time points (between 45 and 195 min apart) within 12 h. On average, participants answered on 54.5 beep-induced occasions (*SD* = 10.2). At each occasion, we assessed momentary affective valence [‘At the moment I feel …’, scale: −3 (‘bad’) to +3 (‘good’)] and the degree of emotion regulation [‘I tried to intensify my pleasant feelings’; scale: 0 (‘not at all’) to +6 (‘very much’)] since the last occasion. In the following, we differentiate between momentary self-reported affective valence during the fMRI task (AffVal_fMRI_) and momentary self-reported affective valence during the ESM phase (AffVal_ESM_).

### MRI acquisition and processing

MRI was performed at the Berlin Center for Advanced Neuroimaging using a 3-T Siemens Tim Trio MRI (Siemens, Erlangen, Germany) with a standard 12-channel head coil. T1-weighted images were acquired with an Magnetization Prepared RApid Gradient Echo (MPRAGE) sequence (TR = 1900 ms, TE = 2.52 ms, FOV = 256 mm, 192 slices, flip angle = 9°, voxel size = 1 mm isotropic). Functional images were acquired using a T2*-weighted gradient-echo echo-planar imaging (EPI) sequence (TR = 2090 ms, TE = 22 ms, flip angle = 90°, FOV = 192 mm, voxel size = 3 mm isotropic). A total of 40 slices of 2.5 mm (0.5 mm gap) were obtained in interleaved order parallel to the anterior-posterior commissure line. A field map (TR = 438, TE_1_ = 5.19 ms, TE_2_ = 7.65 ms, flip angle = 60°, FOV = 192 mm) was acquired (before the EPI sequence) for distortion correction. The experiment was presented on an MR-compatible screen (NordicNeuroLab, Bergen, Norway) using OpenSesame 3.0.6 (Mathôt *et al*., 2012). MR images were processed and analyzed using SPM12 (http://www.fil.ion.ucl.ac.uk/spm/software/spm12/). First, four dummy scans, acquired at the beginning of each run, were excluded. FMRI preprocessing consisted of slice time correction (via interpolation), realignment to the mean EPI, co-registration of the T1-weighted image to the mean EPI, segmentation into three tissue classes (GM, WM, CSF) and normalization to Montreal Neurological Institute (MNI) space (3 mm isotropic voxels) with the IXI555 template (from 555 healthy subjects; www.brain-development.org) plus spatial smoothing (with an 8 mm full-width-at-half-maximum Gaussian kernel) using DARTEL. This kernel size was chosen to parallel the study from which we obtained the VS mask (Rothkirch *et al*., 2014; see below) and because it fulfills the recommendation of at least twice the voxel dimension (Poldrack *et al*., 2011, p. 118). No participant had to be excluded due to head movement (cut-off, >0.3 mm of mean frame displacement; Power *et al*., 2012, 2015).

### Statistical analyses

#### Behavioral analyses

As a manipulation check, we first tested successful emotion regulation during fMRI and in daily life using linear-mixed modeling. Successful up-regulation of positive emotions during fMRI (i.e. higher levels of AffVal_fMRI_ during up-regulation to positive images compared to just watching them and to up-regulation to neutral images) was determined using the post-image AffVal_fMRI_ as the outcome variable with valence (Pos, Neu), instruction (Up, Watch), and their interaction as predictors (for full model, see Supplement 1.3). To determine trial-wise regulation success, the change in affect for each trial was calculated as the difference between the post- and pre-image AffVal_fMRI_. The pre-image rating provides a trial-specific baseline, reflecting within-person changes in affect more directly (Augustine and Hemenover, 2009).

To test successful emotion regulation in daily life, momentary affect at each occasion (AffVal_ESM_) was used as the outcome variable and the degree of emotion regulation as a predictor. To get a better proxy of the ‘change’ in AffVal_ESM_, AffVal_ESM_ at the previous occasion was included as a lagged score as an additional predictor (for full model, see Supplement 1.3). Measures from these analyses were used for hypothesis-specific tests of a relation between neural activation and differences in affect (see below).

#### FMRI—first- and second-level analyses

At the first level, a general linear model was specified for each participant to model the BOLD signal for each condition (using a canonical hemodynamic response function). Data were high-pass filtered (cut-off, 128 s) to remove low-frequency drifts. Autocorrelated residuals were accounted for by an autoregressive model, AR(1). The image (8 s), the affect ratings (exact duration, max. 8 s), the instruction (2 s), the fixation cross (1 s) and the break (4 s) were all modeled with their respective duration as separate regressors. Besides these six regressors of interest, the six motion parameters were entered as regressors of no interest. At the second (i.e. group) level, random effects analysis was performed. According to our hypotheses, region of interest (ROI) analyses of the right and left VS were conducted with a binary mask (total size, 208 voxels). This mask was based on coordinates from nine reward-related studies (Rothkirch *et al*., 2014 for more details), which were pooled and smoothed with a 3D Gaussian kernel of two standard deviations. Statistical parametric maps in the bilateral ROI were family-wise error (FWE)-corrected for multiple comparisons at *P* < 0.05.

VS ROI analyses were complemented by exploratory whole-brain analyses, for which cluster-extent based thresholding was used with *P* < 0.001 (uncorrected) at the voxel and *P* < 0.05 (FWE-corrected) at the cluster level. For each contrast, cluster extent thresholds *k* (ranging from 92 to 113 voxels) were estimated with the ‘SPM Cluster Size Threshold’ tool (version date: 12 January 2016; https://github.com/CyclotronResearchCentre/SPM_ClusterSizeThreshold).

To test for an association between dynamic within-person changes in affect (i.e. trial-by-trial changes in AffVal_fMRI_) and the BOLD signal, parametric analyses were conducted: changes in AffVal_fMRI_ were included as a parametric regressor at the first level, and a one-sample *t*-test was performed at the second.

All resulting t-maps are available on NeuroVault (Gorgolewski *et al*., 2015): to psychologically interpret the results of the exploratory whole-brain analysis in a data-driven way, the respective t-maps were compared (using NeuroVault’s ‘decode’ function) with terms of the online database Neurosynth, which contains activations and associated (psychological, anatomical) labels from 14 371 studies (Yarkoni *et al*., 2011).

All (Pearson) correlations of the links between behavioral and neural measures were outlier-corrected (3 *SD*) and a (two-sided) α-level of 0.05 was used to determine statistical significance.

#### VS activity during up-regulation

To examine whether the VS is particularly activated during the up-regulation to positive images, compared to just watching them and to up-regulating neutral images, the interaction of valence and instruction ([PosUp > PosWatch] > [NeuUp > NeuWatch]), their two main effects (Pos > Neu and Up > Watch) and—given the study’s focus on regulation effects—the simple effects PosUp > PosWatch and NeuUp > NeuWatch were analyzed in the VS.

Next, the hypothesis was tested that increased activation in the VS is related to higher between-person levels of affect when up-regulating positive emotions (i.e. successful up-regulation). For this, VS activity of the PosUp > PosWatch contrast was correlated with person-specific estimates of the random slopes from the linear-mixed model of the behavioral data (positive trials only), which represent AffVal_fMRI_ during PosUp vs. PosWatch.

To test whether increased activation in the VS is related to greater within-person (i.e. trial-by-trial) changes in affect when up-regulating positive emotions, we conducted a parametric analysis with changes in AffVal_fMRI_ for the PosUp condition only (*n* = 60, as three participants showed no variance in their AffVal_fMRI_ in this condition). The use of a parametric regressor to account for within-person changes in affect during emotion regulation follows earlier studies that used a similar approach (e.g. Phan *et al*., 2005).

#### Relating VS activity and up-regulation in daily life

As a behavioral check, we correlated mean levels and variability of affect from the laboratory and from daily life. That is, we extracted person-specific estimates of the random intercepts from our multilevel models of the lab-based and ESM data (leaving out any predictors), reflecting mean levels of AffVal_fMRI_ and AffVal_ESM_, respectively. Additionally, we computed within-person standard deviations of these affect measures. We then calculated the Pearson correlation coefficients between the means and standard deviations, respectively.

To test the hypothesis that greater VS activation occurring when instructed to up-regulate during fMRI is related to higher changes in momentary affect when up-regulating in daily life, person-specific estimates of the random slopes were extracted from the linear-mixed model of the ESM data. These estimates (i.e. each person’s change in AffVal_ESM_ in relation to the degree of up-regulation) were then correlated with VS activity when up-regulating positive emotions (extracted parameter estimates from PosUp > PosWatch).

#### Emotion-related brain activity and its association with affect in daily life

To test which brain regions—beyond the VS—are associated with changes in affect during task performance, an exploratory parametric whole-brain analysis was conducted examining how trial-by-trial changes in AffVal_fRMI_ relate to the BOLD signal. To increase comparability between affect measured in the laboratory and in daily life (no regulation instructions nor information on the affective valence of the exact situation in which affect ratings are provided in the latter), the parametric analysis included changes in affect across all conditions irrespective of instruction or stimulus valence (i.e. all trials of the up-regulate and watch condition, neutral and positive pictures). To explore how neurobehavioral associations in the lab relate to affective experiences in daily life, we extracted the mean parameter estimate for each participant from the parametric modulation analysis across all clusters that showed a significant positive relation with trial-by-trial changes in AffVal_fMRI_. These parameter estimates, reflecting each participant’s strength of the association between changes in AffVal_fMRI_ and BOLD signal across the activated clusters, were then correlated with the person-specific estimates of the random intercepts from the linear-mixed model predicting AffVal_ESM_ (i.e. mean levels of momentary affect in daily life).

## Results

### Behavioral results

**Fig. 2:**
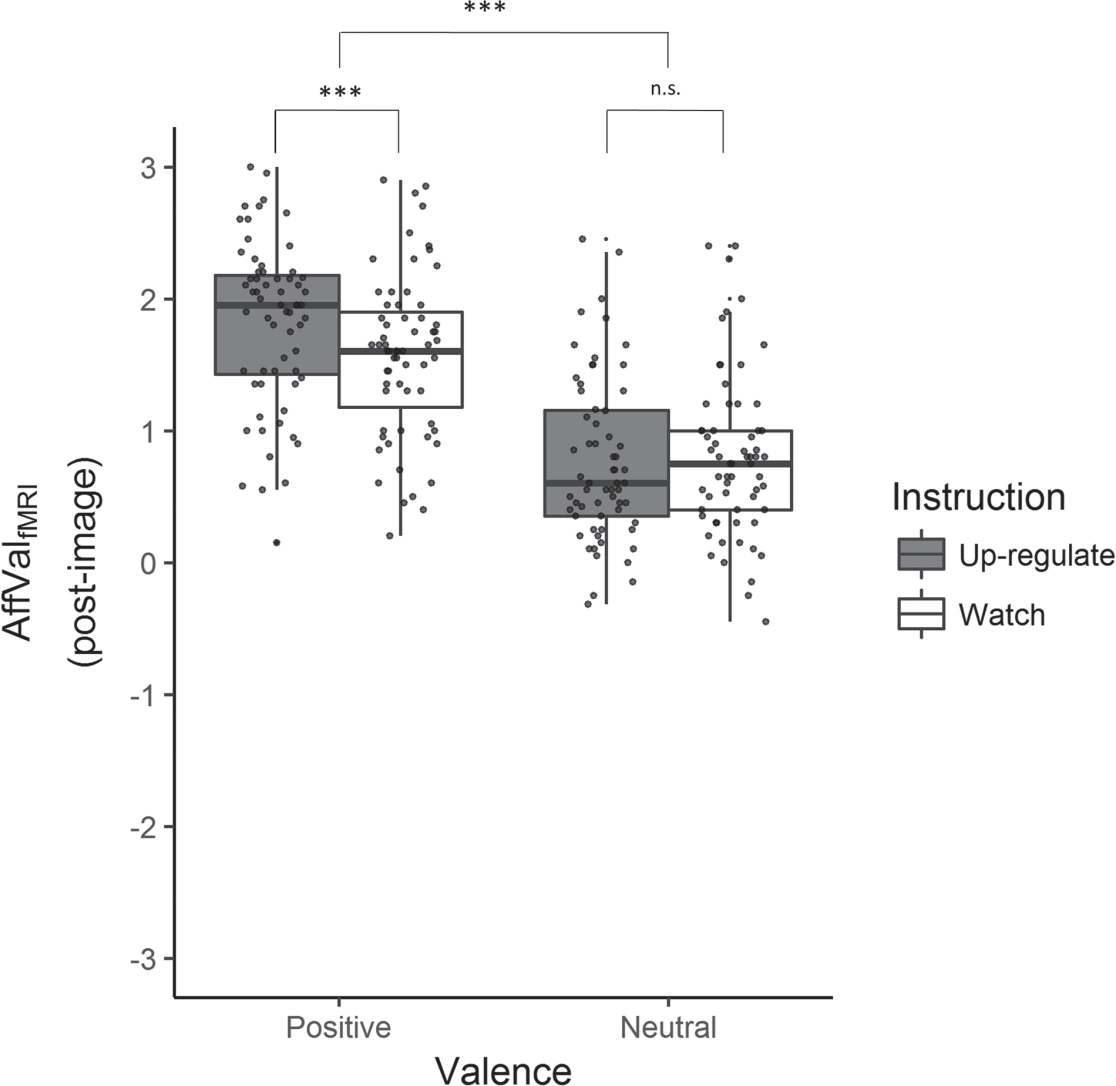
Self-reported affective valence in the emotion regulation task in the fMRI. There was a significant main effect of valence and a significant valence-by-instruction interaction effect. That is, self-reported affective valence (AffValfMRI; post-image ratings) was more positive for positive compared to neutral images and more positive for up-regulating emotions to positive (*vs* passively watching them) compared to neutral images (*vs* passively watching them). No significant difference was observed for up-regulating neutral images *vs* passively watching them. Results are displayed as boxplots with median and first and third quartile. ^***^P < 0.001; n.s., not significant.

For up-regulation during fMRI, a significant main effect of valence, β = 0.76, *P* < 0.001, and interaction effect, β = 0.28, *P* < 0.001, were found, but no significant main effect for instruction, β = −0.04, *P* = 0.53. Following up on the significant interaction, separate analyses were conducted for positive and neutral trials, keeping only instruction as a predictor. This showed that participants successfully up-regulated to positive, β = 0.24, *P* < 0.001, but not to neutral images, β = −0.04, *P* = 0.52, Figure 2; Supplementary Table S2.

In daily life, participants had a greater change in AffVal_ESM_, the more strongly they up-regulated their positive emotions (significant main effect of the degree of emotion regulation, β = 0.29, *P* < 0.001, and AffVal_ESM_ at the previous occasion, β = 0.14, *P* < 0.001; Supplementary Table S3).

### FMRI results

#### VS activity during up-regulation

No significant voxels were found for the interaction ([PosUp > PosWatch] > [NeuUp > NeuWatch]) in the VS, which would have indicated higher activation specifically for the up-regulation to positive images, compared to passively watching them and the up-regulation of neutral images. However, in bilateral VS, main effects of valence ([−12, 3, −9], T = 4.0; [18, 0, −9], T = 4.6) and instruction ([−15, 0, −6], T = 5.44; [15, 3, −3], T = 6.47; [−9, 18, 0], T = 3.13) were significant. Follow-up analyses showed significant activation in the bilateral VS for the simple effects PosUp > PosWatch ([−15, 0, −6], T = 4.42; [15, 0, −6], T = 4.54) and NeuUp > NeuWatch ([−18, 3, −3], T = 4.86; [15, 6, −3], T = 5.92). That is, there was higher activation in the VS while up-regulating to both positive and neutral images, as compared to just watching them.

#### VS activity and between-person differences in self-reported affective valence

**Fig. 3:**
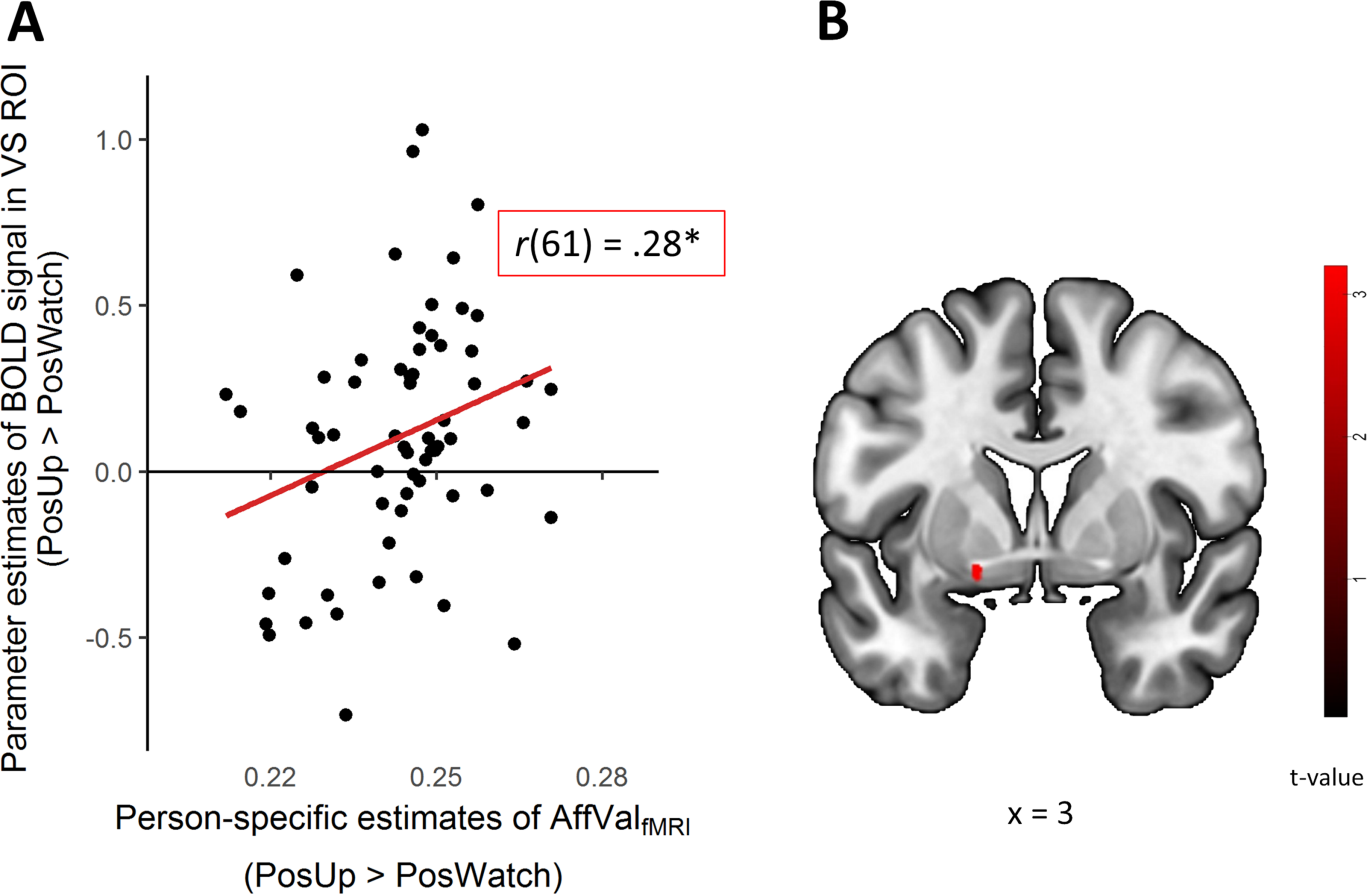
Association of activity in the VS with between-person (i.e. across all trials) and within-person (i.e. trial-by-trial) differences in self-reported affective valence during the fMRI task (AffValfMRI). (A) Increased VS activity (mean activation across the entire ROI) was related to mean differences in AffValfMRI for the upregulation of emotions to positive images (PosUp), compared to passively watching them (PosWatch) and (B) positive association of changes in AffValfMRI in the left VS during PosUp (ROI analysis: [−12, 6, −12], T = 3.39, *P <* 0.05, FWE-corrected). ^*^P < 0.05.

Participants with stronger activation in the VS when up-regulating to positive images (PosUp > PosWatch) also reported more AffVal_fMRI_ across trials, *r*(61) = 0.28, *P* = 0.03; Figure 3A).

**Fig. 4:**
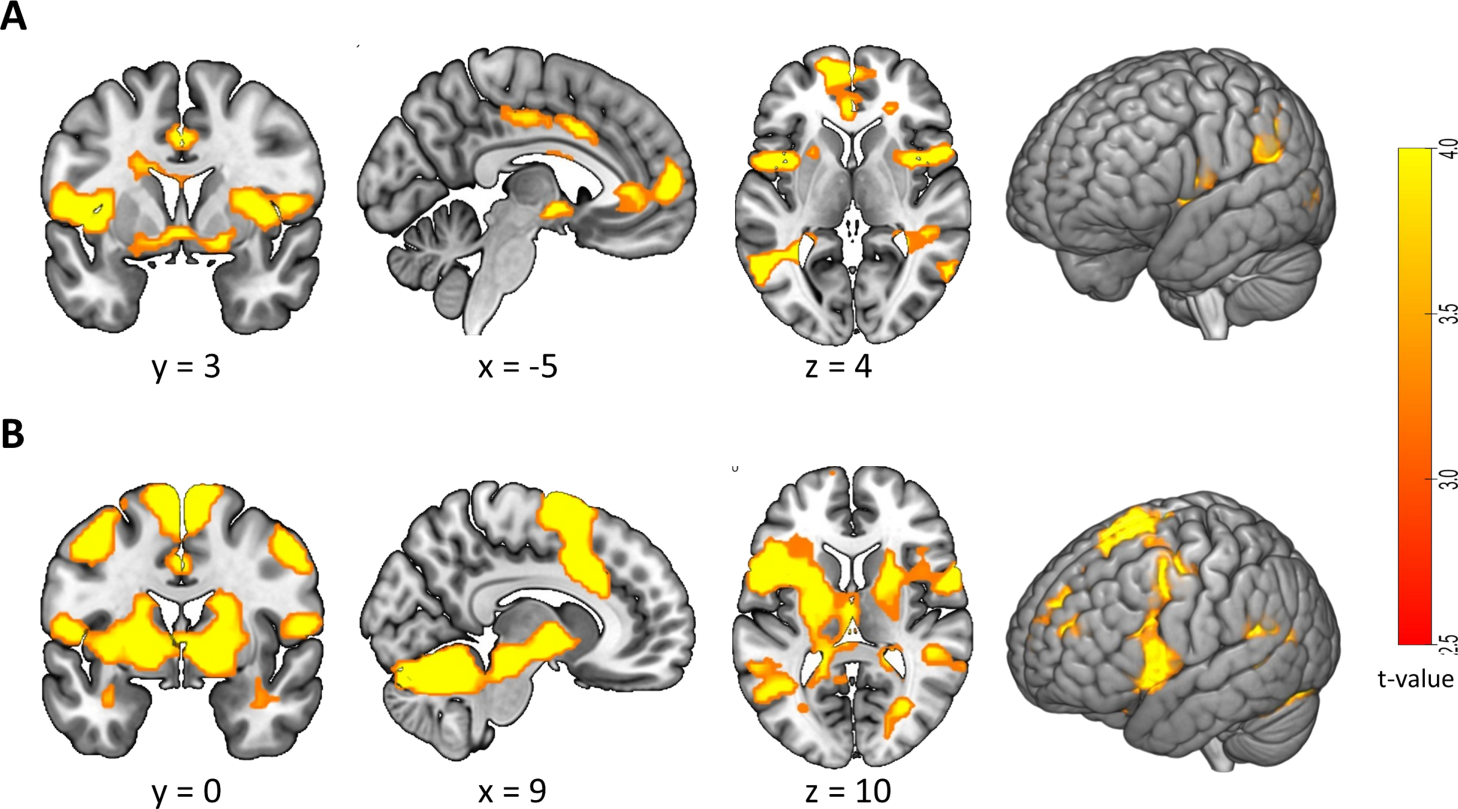
Brain activation in the emotion regulation task (main effects). Regions of increased activation for the (A) main effect of valence (Positive > Neutral) and (B) main effect of instruction (Up > Watch). No significant voxels were found for the interaction. Threshold: *P* < 0.001 (uncorrected) at the voxel and *P* < 0.05 with FWE correction at the cluster level. For details, cf. Table 1. Coordinates are in MNI space.

#### VS activity and within-person changes in affect

Relatively greater trial-by-trial changes in AffVal_fMRI_ were related to increased engagement of the VS during the up-regulation of positive emotions, as shown by parametric increases in the left VS (PosUp condition; [−12, 6, −12], T = 3.39, Figure 3B).

#### Whole-brain activity during up-regulation

In the exploratory whole-brain analysis of increased activation during the up-regulation specifically of positive images (compared to just watching them and to the up-regulation to neutral images, i.e. the interaction of valence and instruction), no voxels survived multiple comparison correction. The main effect of valence (Pos > Neu) showed widespread activation in lateral and medial temporal, frontal and parietal cortices and in subcortical areas (Figure 4A, Table 1). The main effect of instruction (Up > Watch) yielded activation in a large cluster around the left supplementary motor area and in frontal, occipital and cerebellar clusters (Figure 4B, Table 1). Hypoactivation results (i.e. the inverse contrasts) are reported in the Supplement (Supplementary Table S4, Section 1.4, and Supplementary Figure S1).

**Table 1:** Whole-brain analysis for the interaction, main effect of valence and main effect of instruction. For corresponding brain plots, see Figure 4. For the inverse contrasts, see Supplementary Table S4 and Supplementary Figure S1

				MNI coordinates		
Brain regions	Side	k	t	*x*	*y*	*z*
Interaction						
No significant voxels						
Positive > Neutral						
Supramarginal gyrus	R	502	7.70	66	−39	27
Supramarginal gyrus	L	318	7.23	−60	−36	30
Middle temporal gyrus	L	277	6.94	−60	−60	3
Inferior occipital	R	147	6.73	42	−84	−9
Superior frontal gyrus	L	441	5.89	−15	60	3
Precuneus	R	192	5.61	21	−42	12
Insula	L	676	5.55	−42	6	0
Rolandic operculum	R	248	5.34	51	6	6
Midcingulate gyrus	L	339	5.18	−12	−24	42
Up-regulate > Watch						
Supplementary motor area	L	10 731	7.54	−9	15	69
Middle frontal gyrus	R	163	6.69	51	0	51
Calcarine sulcus	R	132	4.40	30	−72	9

*Note.* Clusters labeled according to the anatomical labeling (AAL) atlas (Tzourio-Mazoyer *et al*., 2002). Threshold: *P* < 0.001 (uncorrected) at the voxel level and *P* < 0.05 with FWE correction at the cluster level.

#### Association of whole-brain activity and changes in affect

**Fig. 5:**
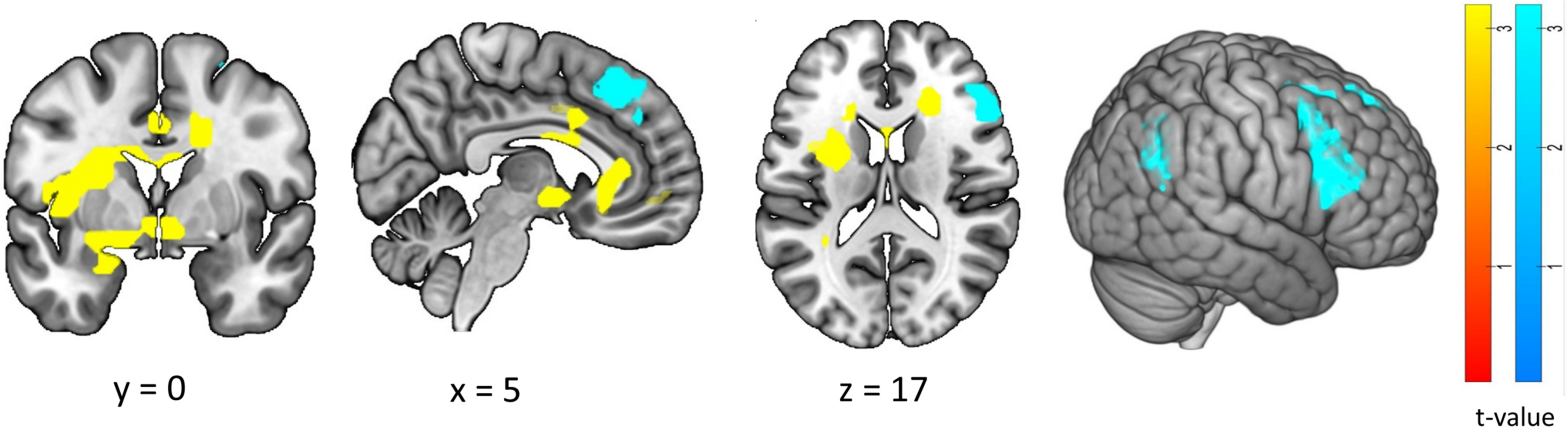
Whole-brain parametric analysis with changes in affect. Regions in which the BOLD signal was positively (yellow) or negatively (blue) related to changes in self-reported affective valence (AffValfMRI) during image presentation in the fMRI task (across all conditions). Threshold: *P* < 0.001 (uncorrected) at the voxel level and *P* < 0.05 with FWE correction at the cluster level. For details, see Table 2.

The exploratory analysis of associations between trial-by-trial changes in AffVal_fMRI_ across all conditions and activation across the whole brain showed significantly positive correlations in widespread regions around medial frontal and subcortical areas and significantly negative correlations in lateral parietal but also in medial and lateral frontal areas, extending into the left insula (Figure 5, Table 2). The Neurosynth analysis mainly associated these regions with the anatomical labels ‘amygdala’, ‘hippocampus’, ‘ventromedial prefrontal cortex (PFC)’ and the psychological concepts ‘arousal’, ‘emotion’ and ‘valence’ for the positive association with changes in affect and with ‘inferior frontal’, ‘parietal’, ‘dorsolateral’ and ‘working memory’, ‘task’ and ‘comprehension’ for the negative association with changes in AffVal_fMRI_ (for a full list of the first 25 entries and their correlation values, see Supplementary Table S5).

### Neurobehavioral associations of up-regulation in fMRI and in daily life

#### Affect in fMRI and in daily life

Participants who had higher means of AffVal_fMRI_ also had higher means of AffVal_ESM_, *r*(61) = 0.31, *P* = 0.01, Supplementary Figure S2. Moreover, greater affect variability (within-person standard deviations) in the laboratory was associated with greater affect variability in daily life, *r*(61) = .37, *P* = 0.003, Supplementary Figure S2.

**Table 2:** Whole-brain parametric analysis with changes in affect. For corresponding brain plots, see Figure 5

				MNI coordinates		
Brain regions	Side	k	t	*x*	*y*	*z*
Increased activation						
Anterior cingulate gyrus	R	1010	5.77	18	33	3
Caudate nucleus	R	210	5.49	6	3	−6
Hippocampus	L	148	5.4	−27	−36	0
Middle occipital gyrus	L	119	4.45	−39	−60	0
Decreased activation						
Middle frontal gyrus	R	765	5.44	45	18	45
Inferior frontal gyrus, pars orbitalis	L	115	4.91	−39	18	−12
Angular gyrus	R	219	4.57	54	−57	33

*Note.* Clusters labeled according to the anatomical labeling (AAL) atlas (Tzourio-Mazoyer *et al.*, 2002). Threshold: *P* < 0.001 (uncorrected) at the voxel level and *P* < 0.05 with FWE correction at the cluster level.

#### Relation between VS activity and up-regulation in daily life

The association between VS activity during the up-regulation of positive emotions (PosUp > PosWatch) during fMRI was not related to the change in AffVal_ESM_ during up-regulation in daily life, *r*(61) = 0.00, *P* = 0.97.

#### Exploring whole-brain activity, changes in affect and affect in daily life

**Fig. 6:**
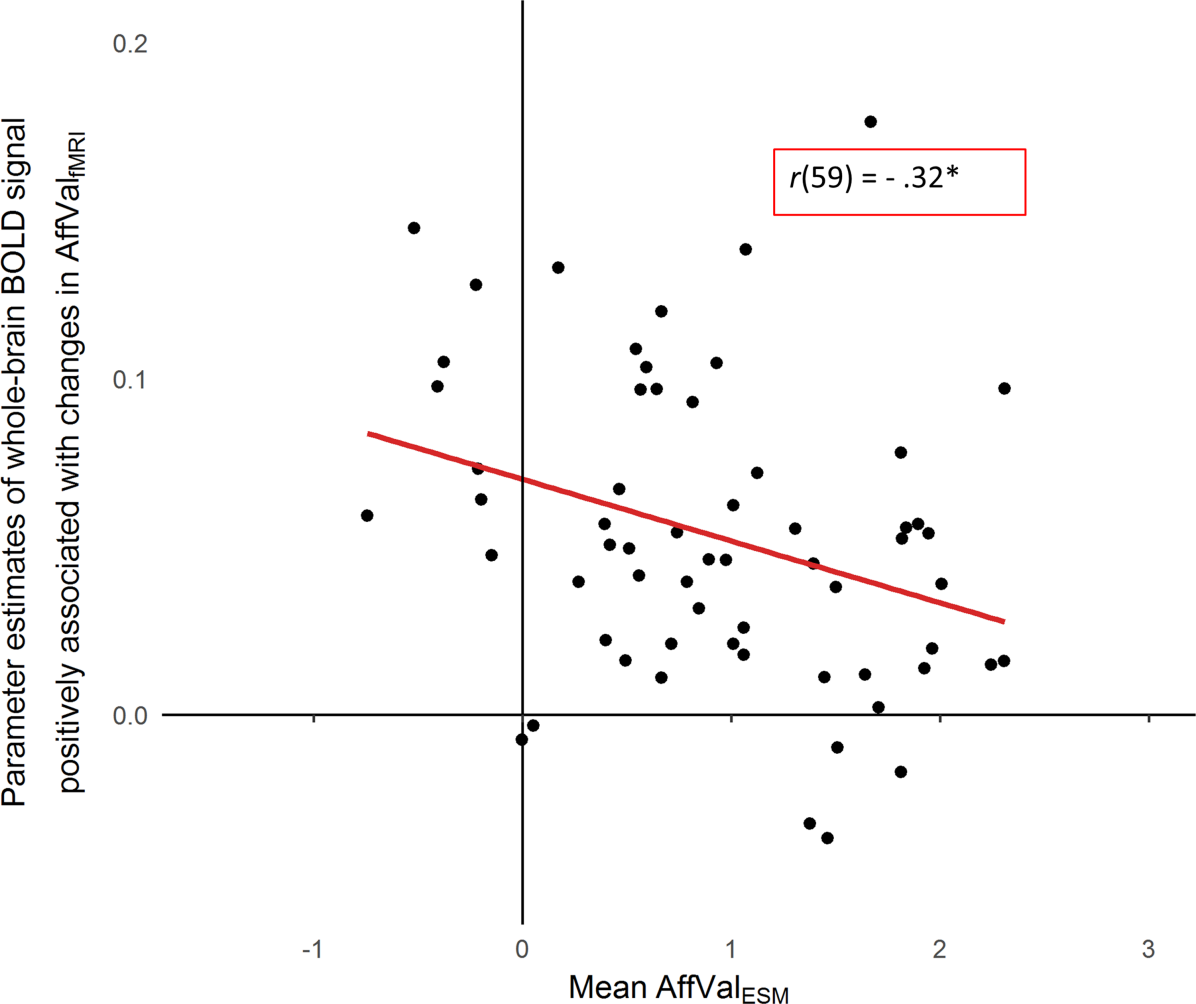
Link between affect in daily life and emotion-related brain activation in the laboratory. Mean self-reported affective valence in daily life (AffValESM) was negatively correlated with the BOLD signal in medial frontal and subcortical emotion-related regions that showed a significant positive association with self-reported affective valence during the fMRI task (AffValfMRI; whole-brain parametric analysis; see Table 2 and yellow clusters in Figure 5). ^*^P < 0.05.

Parameter estimates from the whole-brain parametric analysis of trial-by-trial changes in AffVal_fMRI_ (from all clusters that showed a significant positive association with AffVal_fMRI_, cf. Figure 5) were significantly negatively correlated with mean AffVal_ESM_ in daily life, *r*(59) = −0.32, *P* = 0.01, Figure 6. That is, participants with lower daily affect showed a stronger association between changes in AffVal_fMRI_ and activation in these emotion-related regions.

## Discussion

This study investigated neurobehavioral associations of the up-regulation of positive emotions during fMRI and their relation to emotion regulation and affect in daily life. Specifically, we tested the involvement of the VS in the experience of affect during the up-regulation of positive emotions. We found that VS activation was increased during the up-regulation to images, relative to passively watching them, irrespective of their content’s valence (positive or neutral). For positive images, increased VS activity was related to (i) higher between-person differences in AffVal_fMRI_ and (ii) greater within-person changes in AffVal_fMRI_ during up-regulation. This shows that the VS is not only activated persistently across contexts but also tracks within-person changes in affect—suggesting a central role for the VS in the up-regulation of positive emotions. Against our hypothesis, VS activity was not significantly related to changes in AffVal_ESM_ when up-regulating positive emotions in daily life. However, an exploratory (whole-brain) parametric analysis showed that the lower a participant’s mean AffVal_ESM_ in daily life, the stronger the involvement of a set of medial frontal and subcortical emotion-related brain regions (including the VS) in changing affect during the task in the laboratory.

### Up-regulation to positive and neutral images

We did not find the VS to be uniquely activated during the up-regulation to positive images but also during the up-regulation to neutral images. Behaviorally, however, the up-regulation to neutral images did not change participants’ AffVal_fMRI_. Thus, in addition to the VS representing heightened positive experiences (Kringelbach and Berridge, 2009), it may serve another function during emotion regulation: VS activity may represent the general pursuit of an up-regulation goal (Ochsner *et al*., 2012). This notion is in line with the meta-analytic finding of increased VS activity during the up-regulation (as compared to the down-regulation) of ‘both’ positive and negative emotions (Morawetz *et al*., 2017).

Like a previous study (Greening *et al*., 2014), we found that increased activation in the VS was associated with more positive AffVal_fMRI_ (across trials) when up-regulating positive emotions. Hence, the strength of VS recruitment can be considered a neural indicator of between-person differences in the ability to up-regulate positive emotions. We additionally found that increased activation in the VS was associated with greater moment-to-moment changes in AffVal_fMRI_ during the up-regulation of positive emotions. Thus, the VS seems to be sensitive to varying regulatory efforts that may result from factors such as the specific type (Heiy and Cheavens, 2014) or intensity (Silvers *et al*., 2015b) of the emotion to be regulated.

### Neural responses underlying changes in affect

Our data suggest that other brain regions and networks (in addition to the VS) also reflect changes in affective experiences. The whole-brain parametric analysis showed that changes in AffVal_fMRI_—also an index of successfully up-regulating positive emotions—were associated with activation in several brain regions that have been implicated in affective functioning, such as amygdala, hippocampus, ventromedial PFC and striatum (cf. our Neurosynth decoding results). This finding aligns with the ‘affective workspace hypothesis’ that affective experiences rely on a flexible set of brain regions generally implicated in affective processing rather than on single brain regions representing positivity or negativity (Lindquist *et al*., 2016).

Besides activation in these emotion-related regions, changes in AffVal_fMRI_ were also associated with hypoactivation of a fronto-parietal network, comprising lateral parietal and medial as well as lateral prefrontal cortices, which has previously been related to goal-directed cognition in general (Spreng *et al*., 2010) and the cognitive control of emotions in particular (Ochsner *et al*., 2012). Studied mainly in the context of the down-regulation of negative emotions, this network has repeatedly been shown to be active during cognitive reappraisal (Buhle *et al*., 2014) and associated with within-person changes in ‘negative’ affective experiences (Silvers *et al*., 2015a). In our study, similar prefrontal control regions were relatively ‘less’ recruited with positive changes in AffVal_fMRI_. Two possible explanations for this finding are the following.

First, hypoactivation in these prefrontal regions might indicate that increasing one’s positive affect (e.g. during the up-regulation of positive emotions) is less cognitively challenging and involves less cognitive control (‘less suppression’) of subcortical emotion regions than, for example, the active down-regulation of negative emotions, as suggested previously (Morawetz *et al*., 2017). During the up-regulation of positive emotions, an already existing affective experience is further intensified and regulatory efforts are suggested to be inversely proportional to the intensity of the affective experience to be regulated (Quoidbach *et al.*, 2015). Hence, while the up-regulation of mild positive affect or the down-regulation of negative emotions may require (more) cognitive effort to change an emotional response (e.g. by altering its meaning through reappraisal; Buhle *et al*., 2014), up-regulating (intense) positive emotions may simply mean ‘admitting more’ of an already existing emotional experience. Along these lines, participants are thought to regulate their positive emotions, as compared to regulating their negative emotions, more frequently and more successfully in their daily lives (Heiy and Cheavens, 2014).

Second, the present finding suggests that enhancing momentary affective experiences might initiate distinct processes compared to other forms of emotion regulation. A recent study found hypoactivation in right fronto-parietal regions for the endogenous generation of positive emotions (besides activations in emotion-related regions; Engen *et al*., 2017). Thus, enhancing positive affective experiences may more strongly draw upon emotion generation than on alteration processes, compared to reducing negative affect (Silvers *et al.*, 2015a; see also Supplementary Figure S3 and Supplement 1.5). In sum, the fronto-parietal control network seems to be relevant for the management of both positive and negative affective experiences.

### Relating neurobehavioral associations with emotion regulation and affect in daily life

The hypothesized link between VS activity during up-regulation in fMRI and shifts in momentary affect when up-regulating in daily life was not supported by the data. Also the association between mean levels of self-reported affective valence and affect variability during fMRI and daily life was relatively weak in our study. This may be due to methodological constraints that limit the comparability between measures from the laboratory and the real world. For example, the capacity to change one’s emotional response upon instruction (as tested in the laboratory; Webb *et al*., 2012) possibly differs from the capacity to spontaneously regulate one’s emotions (as usually done in daily life).

Interestingly, in participants with lower mean affect in daily life (i.e. mean AffVal_ESM_), more variance of changes in AffVal_fMRI_ could be explained by activation in a network of emotion-related brain regions (including the VS). This could indicate that the lower one’s affect, the more this ‘core set’ is involved in pro-hedonically changing one’s affective states. Speculatively, such changes could reflect reward-related processes: that is, people feeling worse in daily life have lower expectations of positive events, which leads to higher reward prediction errors and higher mood (Eldar *et al*., 2016; Rutledge *et al*., 2014). Fittingly, a meta-analysis found activation in a similar affective network during the experience of reward as opposed to loss (Liu *et al*., 2011) and recent ESM findings from our group suggest that people with lower well-being benefit more (in terms of their momentary affect) from daily positive events (Grosse Rueschkamp *et al*., 2018).

### Limitations and further directions

There are several limitations: first, as partly discussed above, there are inherent differences between emotion regulation in laboratory-based tasks and in daily life (e.g. standardized stimuli vs. idiosyncratic events or instructed vs. spontaneous emotion regulation). Future studies could aim at establishing a greater similarity between laboratory/fMRI and daily life by, for example, having participants engage in spontaneous rather than instructed emotion regulation during fMRI or by instructing participants to use specific (comparable) strategies in both circumstances.

Second, when investigating affective processes, it is important to consider the timescale at which affective change occurs (Hollenstein *et al*., 2013). During fMRI, changes in affect are measured across seconds, whereas in daily life affective responses are assessed across minutes and hours. Thus, these two measures possibly capture different regulation processes (e.g. mood *vs* affect regulation).

## Conclusion

By enhancing our positive emotional experiences, we can substantially improve the way we feel. This study highlights the relevance of the VS during the up-regulation of positive emotions by showing that not only between-person differences but also dynamic within-person changes in affect are supported by VS activity. The present findings further suggest that the ability to enhance one’s positive experiences might rely less on cognitive control processes, as indicated by the relative hypoactivation in a fronto-parietal network and more on the capacity to endogenously generate emotions. Finally, people who tend to feel worse in daily life show a stronger link between neural activation in emotion-related regions (including the VS) and changes in their affective experiences. Together, these findings emphasize the role of the VS for positive affect and underline the importance of including both laboratory and daily life measures in the study of emotion.

## Conflict of interest

None declared.

## Supplementary Material

fs1_scan-19-025-File011_nsz079

fs2a_scan-19-025-File012_nsz079

fs2b_scan-19-025-File013_nsz079

fs3_scan-19-025-File014_nsz079

scan-19-025-File009_nsz079

scan-19-025-File010_nsz079

## References

[ref1] Araújo D., Davids K., Passos P. (2007). Ecological validity, representative design, and correspondence between experimental task constraints and behavioral setting: comment on Rogers, Kadar, and Costall (2005). Ecological Psychology, 19(1), 69–78 10.1080/10407410709336951.

[ref2] Augustine A.A., Hemenover S.H. (2009). On the relative effectiveness of affect regulation strategies: a meta-analysis. Cognition and Emotion, 23(6), 1181–220 10.1080/02699930802396556.

[ref3] Blood A.J., Zatorre R.J. (2001). Intensely pleasurable responses to music correlate with activity in brain regions implicated in reward and emotion. Proceedings of the National Academy of Sciences of the United States of America, 98(20), 11818–23 10.1073/pnas.191355898. 11573015 PMC58814

[ref4] Buhle J.T., Silvers J.A., Wager T.D., et al. (2014). Cognitive reappraisal of emotion: a meta-analysis of human neuroimaging studies. Cerebral Cortex, 24(11), 2981–90 10.1093/cercor/bht154. 23765157 PMC4193464

[ref5] Doré B.P., Boccagno C., Burr D., et al. (2017). Finding positive meaning in negative experiences engages ventral striatal and ventromedial prefrontal regions associated with reward valuation. Journal of Cognitive Neuroscience, 29(2), 235–44 10.1162/jocn_a_01041. 27626229

[ref6] Eldar E., Niv Y. (2015). Interaction between emotional state and learning underlies mood instability. Nature Communications, 6(1), 6149 10.1038/ncomms7149. PMC5338993 25608088

[ref7] Eldar E., Rutledge R.B., Dolan R.J., Niv Y. (2016). Mood as representation of momentum. Trends in Cognitive Sciences, 20(1), 15–24 10.1016/j.tics.2015.07.010. 26545853 PMC4703769

[ref8] Engen H.G., Kanske P., Singer T. (2017). The neural component-process architecture of endogenously generated emotion. Social Cognitive and Affective Neuroscience, 12(2), 197–211 10.1093/scan/nsw108. 27522089 PMC5390748

[ref9] Gasper K. (2018). Utilizing neutral affective states in research: theory, assessment, and recommendations. Emotion Review, 10(3), 255–66 10.1177/1754073918765660.

[ref10] Giuliani N.R., McRae K., Gross J.J. (2008). The up- and down-regulation of amusement: experiential, behavioral, and autonomic consequences. Emotion, 8(5), 714–9 10.1037/a0013236. 18837622 PMC4138973

[ref11] Gorgolewski K.J., Varoquaux G., Rivera G., et al. (2015). NeuroVault.org: a web-based repository for collecting and sharing unthresholded statistical maps of the human brain. Frontiers in Neuroinformatics, 9, 8 10.3389/fninf.2015.00008. 25914639 PMC4392315

[ref12] Greening S.G., Osuch E.A., Williamson P.C., Mitchell D.G.V. (2014). The neural correlates of regulating positive and negative emotions in medication-free major depression. Social Cognitive and Affective Neuroscience, 9(5), 628–37 10.1093/scan/nst027. 23482626 PMC4014100

[ref13] Grosse Rueschkamp J.M., Kuppens P., Riediger M., Blanke E.S., Brose A. (2018). Higher well-being is related to reduced affective reactivity to positive events in daily life. Emotion, http://doi.org/10.1037/emo0000557. 10.1037/emo0000557 30550304

[ref15] Heiy J.E., Cheavens J.S. (2014). Back to basics: a naturalistic assessment of the experience and regulation of emotion. Emotion, 14(5), 878–91 10.1037/a0037231. 24999913

[ref16] Heller A.S., Fox A.S., Wing E.K., McQuisition K.M., Vack N.J., Davidson R.J. (2015). The neurodynamics of affect in the laboratory predicts persistence of real-world emotional responses. Journal of Neuroscience, 35(29), 10503–9 10.1523/JNEUROSCI.0569-15.2015. 26203145 PMC4510290

[ref17] Hollenstein T., Lichtwarck-Aschoff A., Potworowski G. (2013). A model of socioemotional flexibility at three time scales. Emotion Review, 5(4), 397–405 10.1177/1754073913484181.

[ref18] Jose P.E., Lim B.T., Bryant F.B. (2012). Does savoring increase happiness? A daily diary study. Journal of Positive Psychology, 7(3), 176–87 10.1080/17439760.2012.671345.

[ref19] Kim S.H., Hamann S. (2007). Neural correlates of positive and negative emotion regulation. Journal of Cognitive Neuroscience, 19(5), 776–98 10.1162/jocn.2007.19.5.776. 17488204

[ref21] Kringelbach M.L., Berridge K.C. (2009). Towards a functional neuroanatomy of pleasure and happiness. Trends in Cognitive Sciences, 13(11), 479–87 10.1016/j.tics.2009.08.006. 19782634 PMC2767390

[ref22] Li F., Yin S., Feng P., Hu N., Ding C., Chen A. (2018). The cognitive up- and down-regulation of positive emotion: evidence from behavior, electrophysiology, and neuroimaging. Biological Psychology, 136, 57–66 10.1016/j.biopsycho.2018.05.013. 29787789

[ref23] Lindquist K.A., Satpute A.B., Wager T.D., Weber J., Barrett L.F. (2016). The brain basis of positive and negative affect: evidence from a meta-analysis of the human neuroimaging literature. Cerebral Cortex, 26(5), 1910–22 10.1093/cercor/bhv001. 25631056 PMC4830281

[ref24] Liu X., Hairston J., Schrier M., Fan J. (2011). Common and distinct networks underlying reward valence and processing stages: a meta-analysis of functional neuroimaging studies. Neuroscience & Biobehavioral Reviews, 35(5), 1219–36 10.1016/j.neubiorev.2010.12.012. 21185861 PMC3395003

[ref25] Mathôt S., Schreij D., Theeuwes J. (2012). OpenSesame: an open-source, graphical experiment builder for the social sciences. Behavior Research Methods, 44(2), 314–24 10.3758/s13428-011-0168-7. 22083660 PMC3356517

[ref26] Morawetz C., Bode S., Derntl B., Heekeren H.R. (2017). The effect of strategies, goals and stimulus material on the neural mechanisms of emotion regulation: a meta-analysis of fMRI studies. Neuroscience & Biobehavioral Reviews, 72, 111–28 10.1016/j.neubiorev.2016.11.014. 27894828

[ref27] Moutsiana C., Fearon P., Murray L., et al. (2014). Making an effort to feel positive: insecure attachment in infancy predicts the neural underpinnings of emotion regulation in adulthood. Journal of Child Psychology and Psychiatry, and Allied Disciplines, 55(9), 999–1008 10.1111/jcpp.12198. PMC4263234 24397574

[ref28] Ochsner K.N., Silvers J.A., Buhle J.T. (2012). Functional imaging studies of emotion regulation: a synthetic review and evolving model of the cognitive control of emotion. Annals of the New York Academy of Sciences, 1251(1), E1–24 10.1111/j.1749-6632.2012.06751.x. 23025352 PMC4133790

[ref29] Phan K.L., Fitzgerald D.A., Nathan P.J., Moore G.J., Uhde T.W., Tancer M.E. (2005). Neural substrates for voluntary suppression of negative affect: a functional magnetic resonance imaging study. Biological Psychiatry, 57(3), 210–9 10.1016/j.biopsych.2004.10.030. 15691521

[ref30] Poldrack R.A., Mumford J.A., Nichols T.E. (2011). Handbook of Functional MRI Data Analysis, Cambridge, UK: Cambridge University Press.

[ref31] Power J.D., Barnes K.A., Snyder A.Z., Schlaggar B.L., Petersen S.E. (2012). Spurious but systematic correlations in functional connectivity MRI networks arise from subject motion. NeuroImage, 59(3), 2142–54 10.1016/j.neuroImage.2011.10.018. 22019881 PMC3254728

[ref32] Power J.D., Schlaggar B.L., Petersen S.E. (2015). Recent progress and outstanding issues in motion correction in resting state fMRI. NeuroImage, 105, 536–51 10.1016/j.neuroimage.2014.10.044. 25462692 PMC4262543

[ref32a] Quoidbach J., Mikolajczak M., Gross J.J. (2015). Positive interventions: An emotion regulation perspective. Psychological bulletin, 141(3), 655–693 10.1037/a0038648. 25621978

[ref33] Riediger M., Schmiedek F., Wagner G.G., et al. (2009). Seeking pleasure and seeking pain: differences in prohedonic and contra-hedonic motivation from adolescence to old age. Psychological Science, 20(12), 1529–35 10.1111/j.1467-9280.2009.02473.x. 19891749

[ref33a] Rothkirch M., Schmack K., Deserno L., Darmohray D., Sterzer P. (2014). Attentional modulation of reward processing in the human brain. Human brain mapping, 35(7), 3036–3051 10.1002/hbm.22383. 24307490 PMC6869517

[ref34] Rutledge R.B., Skandali N., Dayan P., Dolan R.J. (2014). A computational and neural model of momentary subjective well-being. Proceedings of the National Academy of Sciences of the United States of America, 111(33), 201407535 10.1073/pnas.1407535111. PMC4143018 25092308

[ref35] Sabatinelli D., Bradley M.M., Lang P.J., Costa V.D., Versace F. (2007). Pleasure rather than salience activates human nucleus accumbens and medial prefrontal cortex. Journal of Neurophysiology, 98(3), 1374–9 10.1152/jn.00230.2007. 17596422

[ref36] Schultz W., Dayan P., Montague P.R. (1997). Neural substrate of prediction and reward. Science, 275(5306), 1593–9 10.1126/science.275.5306.1593. 9054347

[ref37] Silvers J.A., Wager T.D., Weber J., Ochsner K.N. (2015a). The neural bases of uninstructed negative emotion modulation. Social Cognitive and Affective Neuroscience, 10(1), 10–8 10.1093/scan/nsu016. 24493847 PMC4994839

[ref38] Silvers J.A., Weber J., Wager T.D., Ochsner K.N. (2015b). Bad and worse: neural systems underlying reappraisal of high-and low-intensity negative emotions. Social Cognitive and Affective Neuroscience, 10(2), 172–9 10.1093/scan/nsu043. 24603024 PMC4321618

[ref39] Spreng R.N., Stevens W.D., Chamberlain J.P., Gilmore A.W., Schacter D.L. (2010). Default network activity, coupled with the frontoparietal control network, supports goal-directed cognition. NeuroImage, 53(1), 303–17 10.1016/j.neuroimage.2010.06.016. 20600998 PMC2914129

[ref40] Tzourio-Mazoyer N., Landeau B., Papathanassiou D., et al. (2002). Automated anatomical labeling of activations in SPM using a macroscopic anatomical parcellation of the MNI MRI single-subject brain. NeuroImage, 15(1), 273–89 10.1016/nimg.2001.0978. 11771995

[ref41] Vrticka P., Sander D., Vuilleumier P. (2011). Effects of emotion regulation strategy on brain responses to the valence and social content of visual scenes. Neuropsychologia, 49(5), 1067–82 10.1016/j.neuropsychologia.2011.02.020. 21345342

[ref42] Wager T.D., Davidson M.L., Hughes B.L., Lindquist M.A., Ochsner K.N. (2008). Prefrontal-subcortical pathways mediating successful emotion regulation. Neuron, 59(6), 1037–50 10.1016/j.neuron.2008.09.006. 18817740 PMC2742320

[ref43] Webb T.L., Miles E., Sheeran P. (2012). Dealing with feeling: a meta-analysis of the effectiveness of strategies derived from the process model of emotion regulation. Psychological Bulletin, 138(4), 775–808 10.1037/a0027600. 22582737

[ref44] Wessa M., Kanske P., Neumeister P., Bode K., Heissler J., Schönfelder S. (2010). EmoPics: subjektive und psychophysiologische evaluation neuen bildmaterials für die klinisch-bio-psychologische forschung [EmoPics: subjective and psychophysiological evaluation of new image material for clinical- bio- psychological research]. Zeitschrift für Klinische Psychologie und Psychotherapie, 39(Suppl. 1), 77.

[ref45] Yarkoni T., Poldrack R.A., Nichols T.E., Van Essen D.C., Wager T.D. (2011). Large-scale automated synthesis of human functional neuroimaging data. Nature Methods, 8(8), 665–70 10.1038/nmeth.1635. 21706013 PMC3146590

