# Supplementary figures and images for: Neural correlates of up-regulating positive emotions in fMRI and their link to affect in daily life
Source: Soc Cogn Affect Neurosci. 2019 Oct 31;14(10):1049–59. doi: 10.1093/scan/nsz079 (PMC7053268; doi:10.1093/scan/nsz079)

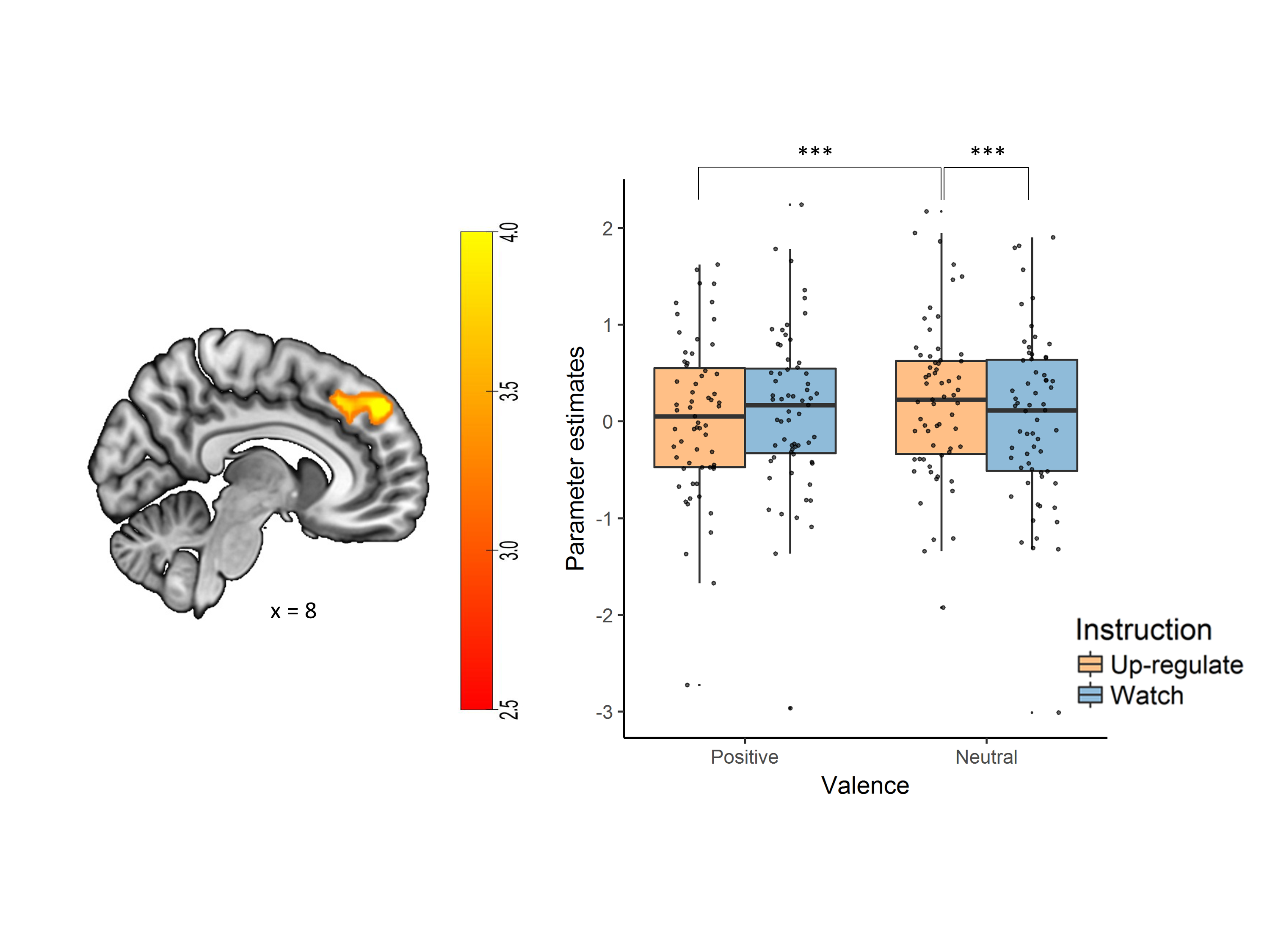

Supplement: fs1_scan-19-025-File011_nsz079 [file fs1_scan-19-025-file011_nsz079.png]

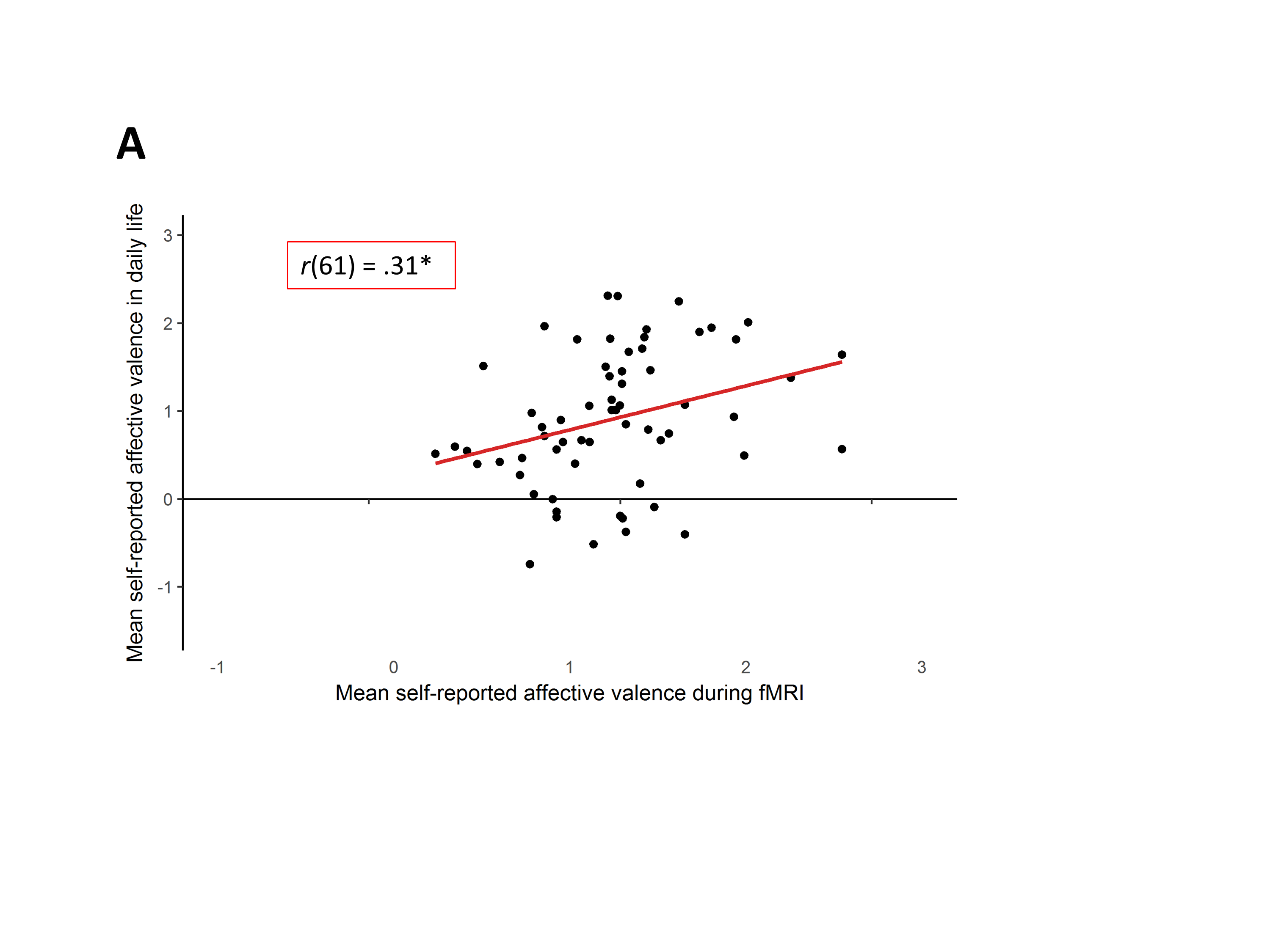

Supplement: fs2a_scan-19-025-File012_nsz079 [file fs2a_scan-19-025-file012_nsz079.png]

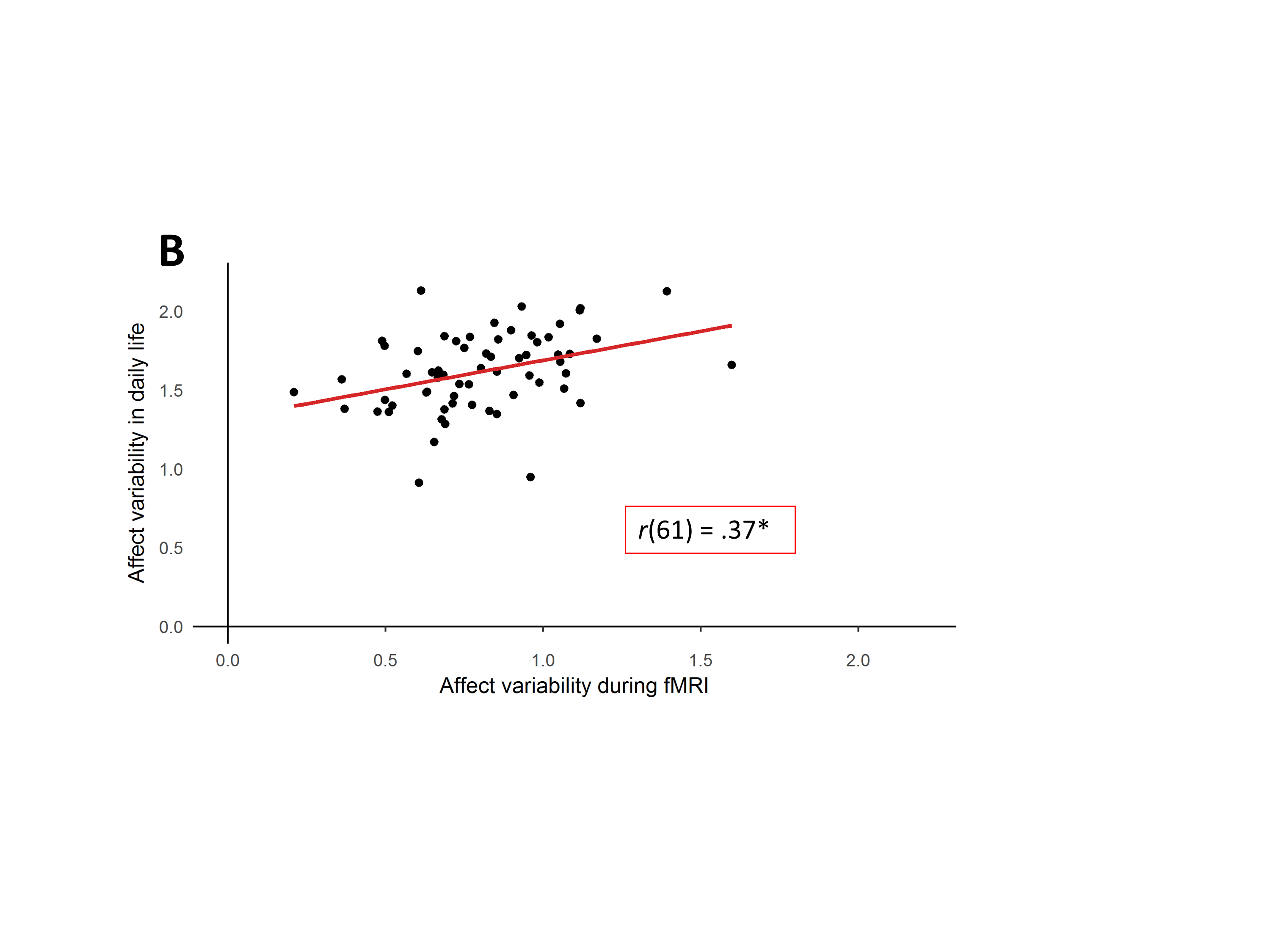

Supplement: fs2b_scan-19-025-File013_nsz079 [file fs2b_scan-19-025-file013_nsz079.png]

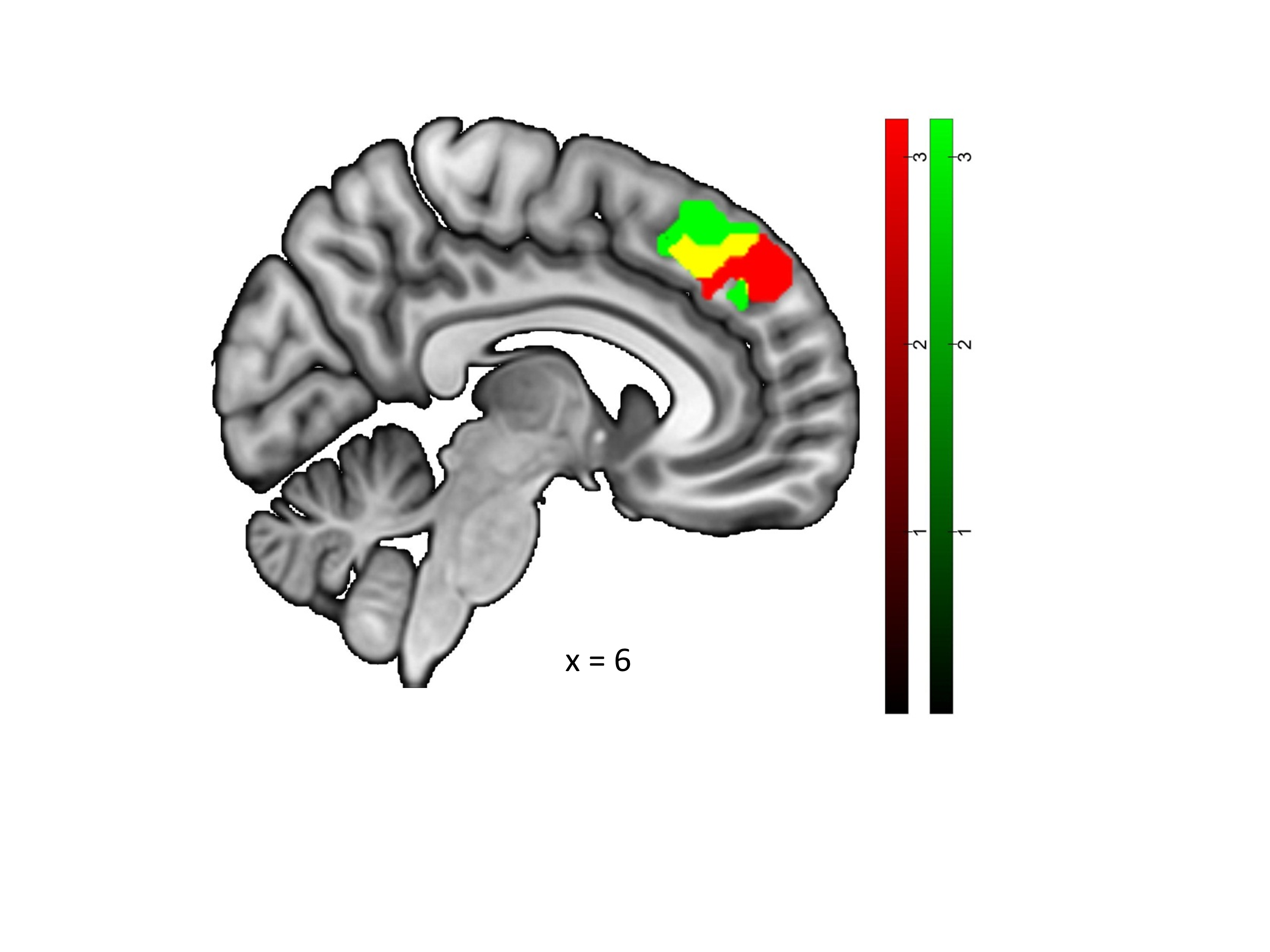

Supplement: fs3_scan-19-025-File014_nsz079 [file fs3_scan-19-025-file014_nsz079.png]
