# Supplementary material for: Neural correlates of up-regulating positive emotions in fMRI and their link to affect in daily life
Source: Soc Cogn Affect Neurosci. 2019 Oct 31;14(10):1049–59. doi: 10.1093/scan/nsz079 (PMC7053268; doi:10.1093/scan/nsz079)
Supplement: scan-19-025-File009_nsz079 [file scan-19-025-file009_nsz079.docx]

**Supplementary material**

1. Methods
   1. *Questionnaires*

Big-Five Inventory (Lang & Lüdtke, 2005)

Satisfaction with Life Scale (Diener, Emmons, Larsen, & Griffin, 1985)

Positive and Negative Affect Scale (Watson, Clark, & Tellegen, 1988)

Cognitive Emotion Regulation Questionnaire (Garnefski, Kraaij, & Spinhoven, 2001)

WHO-5 questionnaire (World Health Organization, 1998)

Emotion Regulation Profile-Revised (Nelis, Quoidbach, Hansenne, & Mikolajczak, 2011)

Dispositional Positive Emotion Scales (Shiota, Keltner, & John, 2006)

Hypomanic Personality Scale (Eckblad & Chapman, 1986)

- 1. *Emotion regulation instructions during fMRI*

*original German wording*

„Willkommen bei dieser Studie.

Sie werden verschiedene Bilder sehen, die unterschiedliche Emotionen hervorrufen können. Vor jedem Bild erhalten Sie eine Anleitung, was Sie tun sollen. Diese lautet entweder 'VERSTÄRKEN oder 'ANSEHEN'.

Wenn Sie die Anleitung 'VERSTÄRKEN' sehen, versuchen Sie bitte, willentlich Ihre Gefühle zu verstärken, die Sie haben, wenn Sie die Bilder sehen, und welche die dargestellte Szene in Ihnen auslöst. Versuchen Sie also, Ihre Gefühle so intensiv wie möglich zu erleben.

Wenn die Anleitung 'ANSEHEN' lautet, möchten wir Sie bitten, Ihre Gedanken und Gefühle einfach kommen und gehen zu lassen, so wie das natürlicherweise passiert.

Vor und nach jedem Bild werden Sie gefragt, wie Sie sich gerade fühlen. Bitte bewerten Sie Ihre Gefühle auf einer Skala von +3 (gut) bis -3 (schlecht), indem Sie mit der linken und rechten Pfeiltaste das entsprechende Kästchen wählen und dann mit der unteren Pfeiltaste Ihre Antwort einloggen.

Bitte antworten Sie spontan ohne langes Nachdenken. Sie haben max. 10 Sekunden Zeit, um eine Antwort zu geben.

Nach einer kurzen Pause werden Sie dann ein weiteres Mal nach Ihren Gefühlen gefragt. Wenn Sie noch Fragen haben, stellen Sie diese bitte jetzt dem Versuchsleiter.“

*Free English translation*

„Welcome to this study.

You will see different images that can evoke different emotions. Before each image you will receive an instruction about what you should do. This can be either “INCREASE” or “WATCH.”

If you see the instruction „INCREASE,” please try to intentionally increase the feelings that you experience while viewing the images and that the depicted scene evoke in you. That is, try to experience your feelings as intensely as possible.

If the instruction is „WATCH,“ we kindly ask you to just let your feelings and thoughts come and go, as they naturally occur.

Before and after each image you will be asked how you currently feel. Please rate your feelings on a scale from + 3 (good) to - 3 (bad) by choosing the corresponding box with the left and right button and then confirming your answer with the middle button.

Please answer spontaneously without thinking for too long. You have maximally 10 seconds to give an answer.

After a short break you will be asked once more about your feelings. If you have questions, please ask the experimenter now.“

- 1. *Behavioral analyses*

The following multilevel models were carried out to test the successful up-regulation of positive emotions during the emotion regulation task during fMRI and in daily life

1. Emotion regulation during fMRI

(Level 1)

AffVal_fMRI_ *_ti_* = β_0_*_i_* + β_1_*_i_* × (valence*_ti_*) + β_2_*_i_* × (instruction*_ti_*) + β_3_*_i_* × (valence × instruction*_ti_*) + r*_ti_*

(Level 2)

β_0_*_i_* = γ_00_ + µ_0_*_i_*

β_1_*_i_* = γ_10_ + µ_1_*_i_*

β_2_*_i_* = γ_20_ + µ_2_*_i_*

β_3_*_i_* = γ_30_ + µ_3_*_i_*

Momentary self-reported affective valence during the fMRI task (AffVal_fMRI_) of person *i* on trial *t* is predicted by an intercept β_0_*_i_*, the valence of the image β_1_*_i_*, the regulation instruction of that trial β_2_*_i_*, and the interaction of valence and instruction β_3_*_i_*. The average within-person intercept and relationships are represented by the parameters γ_00_–γ_30_. The intercept β_0_*_i_* and slope parameters, β_1_*_i_* – β_3_*_i_*, were allowed to vary across individuals by including the random effects µ_0_*_i_* – µ_3_*_i_*.

1. Emotion regulation in daily life

(Level 1)

AffVal_ESM_ *_ti_* = β_0_*_i_* + β_1_*_i_* × (affect at previous occasion*_ti_*) + β_2_*_i_* × (emotion regulation*_ti_*) + r*_ti_*

(Level 2)

β_0_*_i_* = γ_00_ + µ_0_*_i_*

β_1_*_i_* = γ_10_ + µ_1_*_i_*

β_2_*_i_* = γ_20_ + µ_2_*_i_*

Momentary self-reported affective valence during the ESM phase (AffVal_ESM_) of person *i* on occasion *t* is predicted by an intercept β_0_*_i_*, AffVal_ESM_ at the previous occasion β_1_*_i_*, and the degree of emotion regulation on that occasion β_2_*_i_*. The average within-person intercept and relationships are represented by the parameters γ_00_–γ_20_. The intercept β_0_*_i_* and slope parameters, β_1_*_i_* –β_2_*_i_*, were allowed to vary across individuals by including the random effects µ_0_*_i_* –µ_2_*_i_*.

- 1. *Hypoactivation of interaction: Follow-up analyses*

Extracted parameter estimates from the cluster [6, 48, 42] showed a significant valence by instruction interaction, *F*(1, 62) = 19.5, *p* < .001. Pairwise comparisons showed a significant difference between NeuUp (mean *b* =0.21) and PosUp (mean *b* = 0.04), *t*(62)= - 4.57, *p* < .001, as well as between NeuUp and NeuWatch (mean *b* = 0.04), *t*(62) = 4.05, *p* < .001, Figure 3, suggesting that activation in this cluster was driven by *increased* activation in NeuUp.

- 1. *Hypoactivation of interaction and whole-brain parametric analysis*

In its medial part, the fronto-parietal network overlapped with the *hypoactivation* of the dorsomedial prefrontal cortex (dmPFC) cluster found in the interaction of valence and instruction (see above and Figure S3 for the overlap). As this cluster was solely hypoactivated during the up-regulation of positive emotions, this further supports the idea that successful up-regulation of positive emotions at least partly depends on the hypoactivation of prefrontal control systems. However, if one understands the hypoactivation of the dmPFC cluster as a significant *increase* in activation for the up-regulation to neutral images, this cluster could indicate that, similar to the regulation of negative emotions, regulatory efforts to neutral images require auxiliary cognitive functions (Miller & Cohen, 2001). However, these seem to be dissociated from changes in affect, as indicated by the behavioral data and the whole-brain parametric analysis.

**References**

Diener, E., Emmons, R., Larsen, R. J., & Griffin, S. (1985). The Satisfaction with Life scale. *Journal of Personality Assessment*, *49*(1), 71–75.

Eckblad, M., & Chapman, L. J. (1986). Development and validation of a scale for hypomanic personality. *Journal of Abnormal Psychology*, *95*(3), 214.

Garnefski, N., Kraaij, V., & Spinhoven, P. (2001). Negative life events, cognitive emotion regulation and emotional problems. *Personality and Individual Differences*, *30*(8), 1311–1327. http://doi.org/10.1016/S0191-8869(00)00113-6

Lang, F. R., & Lüdtke, O. (2005). Der Big Five-Ansatz der Persönlichkeitsforschung: Instrumente und Vorgehen [The Big-Five approach in perosnality research: Methods and procedures]. In S. Schumann (Ed.), *Persönlichkeit: Eine vergessene Größe der empirischen Sozialforschung* (pp. 29–39). Wiesbaden: VS Verlag für Sozialwissenschaften.

Miller, E. K., & Cohen, J. D. (2001). An integrative theory of prefrontal cortex function. Annual Review of Neuroscience, *24*(1), 167–202. http://doi.org/10.1146/annurev.phyto.41.052002.095656

Morawetz, C., Bode, S., Derntl, B., & Heekeren, H. R. (2017). The effect of strategies, goals and stimulus material on the neural mechanisms of emotion regulation: A meta-analysis of fMRI studies. *Neuroscience & Biobehavioral Reviews*, *72*, 111–128. http://doi.org/10.1016/j.neubiorev.2016.11.014

Nelis, D., Quoidbach, J., Hansenne, M., & Mikolajczak, M. (2011). Measuring individual differences in emotion regulation: The Emotion Regulation Profile-Revised (ERP-R). *Psychologica Belgica*, *51*(1): 49. http://doi.org/10.5334/pb-51-1-49

Shiota, M. N., Keltner, D., & John, O. P. (2006). Positive emotion dispositions differentially associated with Big Five personality and attachment style. *Journal of Positive Psychology*, *1*(2), 61–71. http://doi.org/10.1080/17439760500510833

Watson, D., Clark, L. A., & Tellegen, A. (1988). Development and validation of brief measures of positive and negative affect: The PANAS scales. *Journal of Personality and Social Psychology*, *54*(6), 1063–1070. http://doi.org/10.1037/0022-3514.54.6.1063

World Health Organization. (1998). Info package: Mastering depression in primary care, version2.2. *Copenhagen: WHO, Regional Office for Europe*.
