# Supplementary material for: Neural correlates of up-regulating positive emotions in fMRI and their link to affect in daily life
Source: Soc Cogn Affect Neurosci. 2019 Oct 31;14(10):1049–59. doi: 10.1093/scan/nsz079 (PMC7053268; doi:10.1093/scan/nsz079)
Supplement: scan-19-025-File010_nsz079 [file scan-19-025-file010_nsz079.docx]

| Table 1. | |  |  |  |  |  |  |  |
| --- | --- | --- | --- | --- | --- | --- | --- | --- |
| *Whole-brain analysis for the interaction, main effect of valence, and main effect of instruction. For corresponding brain plots, see Figure 4. For the inverse contrasts, see Table S4 and Figure S1.* | | | | | | | | |
|  | | |  |  |  | MNI coordinates | | |
| Brain regions | | | Side | k | t | *x* | *y* | *z* |
|  | | |  |  |  |  |  |  |
| Interaction | | |  |  |  |  |  |  |
|  | *No significant voxels* | |  |  |  |  |  |  |
| Positive > Neutral | | |  |  |  |  |  |  |
|  | Supramarginal gyrus | | R | 502 | 7.70 | 66 | -39 | 27 |
|  | Supramarginal gyrus | | L | 318 | 7.23 | -60 | -36 | 30 |
|  | Middle temporal gyrus | | L | 277 | 6.94 | -60 | -60 | 3 |
|  | Inferior occipital | | R | 147 | 6.73 | 42 | -84 | -9 |
|  | Superior frontal gyrus | | L | 441 | 5.89 | -15 | 60 | 3 |
|  | Precuneus | | R | 192 | 5.61 | 21 | -42 | 12 |
|  | Insula | | L | 676 | 5.55 | -42 | 6 | 0 |
|  | Rolandic operculum | | R | 248 | 5.34 | 51 | 6 | 6 |
|  | Midcingulate gyrus | | L | 339 | 5.18 | -12 | -24 | 42 |
| Up-regulate > Watch | | |  |  |  |  |  |  |
|  | Supplementary motor area | | L | 10731 | 7.54 | -9 | 15 | 69 |
|  | Middle frontal gyrus | | R | 163 | 6.69 | 51 | 0 | 51 |
|  | Calcarine sulcus | | R | 132 | 4.40 | 30 | -72 | 9 |
| *Note.* Clusters labeled according to the anatomical labeling (AAL) atlas (Tzourio-Mazoyer et al., 2002). Threshold: *p* < .001 (uncorrected) at the voxel level, and *p* < .05 with FWE correction at the cluster level. | | | | | | | | |

| Table 2. | |  |  |  |  |  |  |  |
| --- | --- | --- | --- | --- | --- | --- | --- | --- |
| *Whole-brain parametric analysis with changes in affect. For corresponding brain plots, see Figure 5.* | | | | | | | | |
|  | | |  |  |  | MNI coordinates | | |
| Brain regions | | | Side | k | t | *x* | *y* | *z* |
|  | | |  |  |  |  |  |  |
| Increased activation | | |  |  |  |  |  |  |
|  | Anterior cingulate gyrus | | R | 1010 | 5.77 | 18 | 33 | 3 |
|  | Caudate nucleus | | R | 210 | 5.49 | 6 | 3 | -6 |
|  | Hippocampus | | L | 148 | 5.4 | -27 | -36 | 0 |
|  | Middle occipital gyrus | | L | 119 | 4.45 | -39 | -60 | 0 |
| Decreased activation | | |  |  |  |  |  |  |
|  | Middle frontal gyrus | | R | 765 | 5.44 | 45 | 18 | 45 |
|  | Inferior frontal gyrus, pars orbitalis | | L | 115 | 4.91 | -39 | 18 | -12 |
|  | Angular gyrus | | R | 219 | 4.57 | 54 | -57 | 33 |
| *Note.* Clusters labeled according to the anatomical labeling (AAL) atlas (Tzourio-Mazoyer et al., 2002). Threshold: *p* < .001 (uncorrected) at the voxel level, and *p* < .05 with FWE correction at the cluster level. | | | | | | | | |

| Table S1. | | | |  | | |  |
| --- | --- | --- | --- | --- | --- | --- | --- |
| *Descriptives of task compliance and strategy use during the emotion- regulation task* | | | | | | | |
|  | | | | | *M* | *SD* | |
| Task compliance | | | | |  |  | |
|  | |  | “I tried to intensify my positive feelings” | | 4.7 | 1.18 | |
| Emotion regulation strategies | | | | |  |  | |
|  | |  | Behavioral display *(“I expressed my positive feelings”)* | | 4.61 | 1.56 | |
|  | |  | Attentional deployment *(“I deliberately paid attention to my positive feelings”)* | | 4.74 | 1.06 | |
|  |  | | Cognitive change *(“I placed myself in the situation to experience it more strongly”)* | | 4.93 | 1.8 | |
|  |  | | Positive mental time travel *(“I reminded myself of a similar situation that I have been in or tried to imagine I would experience a similar situation in the future”)* | | 5.21 | 1.23 | |
| *Note.* Scale: 0 (“not at all”) to +6 (“very much”) | | | | | | | |

| Table S2. | | | |  | | | | |  |
| --- | --- | --- | --- | --- | --- | --- | --- | --- | --- |
| *Results from the linear mixed-effects model predicting self-reported affective valence* (AffVal_fMRI_) *and change in* AffVal_fMRI_ *during the fMRI task* | | | | | | | | | |
|  | | | | | Self-reported AffVal_fMRI_  (post-image rating) | | Change in AffVal_fMRI_  (post – pre-image rating) | | |
| Variable | | | | | Estimate | *SE* | Estimate | *SE* | |
| Fixed Effects | | | | |  |  |  |  | |
|  | |  | Intercept | | 0.81*** | 0.08 | -0.30*** | 0.06 | |
|  | |  | Valence | | 0.76*** | 0.07 | 0.79*** | 0.09 | |
|  | |  | Instruction | | -0.04 | 0.06 | -0.09 | 0.06 | |
|  | |  | Valence x Instruction | | 0.28*** | 0.08 | 0.29** | 0.09 | |
| Random Effects | | | | |  |  |  |  | |
|  | Within-person | | | | 1.34 |  | 1.94 |  | |
|  | Between-person | | | |  |  |  |  | |
|  |  | | Intercept | | 0.32 |  | 0.10 |  | |
|  |  | | Valence slope | | 0.19 |  | 0.30 |  | |
|  |  | | Instruction slope | | 0.07 |  | 0.05 |  | |
|  |  | | Valence x Instruction slope | | 0.12 |  | 0.14 |  | |
| *** *p* < .001 ** *p* < .01 | | | | | | |  | | |

| Table S3. | | | |  | | |  |
| --- | --- | --- | --- | --- | --- | --- | --- |
| *Results from the linear mixed-effects model predicting self-reported affective valence* AffVal_ESM_ *when up-regulating positive emotions in daily life* | | | | | | | |
|  | | | | | AffVal_ESM_ | | |
| Variable | | | | | Estimate | *SE* | |
| Fixed Effects | | | | |  |  | |
|  | |  | Intercept | | 0.92*** | 0.11 | |
|  | |  | AffVal_ESM_ at previous occasion | | 0.14*** | 0.02 | |
|  | |  | Degree of emotion regulation | | 0.29*** | 0.03 | |
| Random Effects | | | | |  |  | |
|  | Within-person | | | | 2.37 |  | |
|  | Between-person | | | |  |  | |
|  |  | | Intercept | | 0.69 |  | |
|  |  | | AffVal_ESM_ at previous occasion slope | | 0.01 |  | |
|  |  | | Degree of emotion regulation slope | | 0.02 |  | |
| *** *p* < .001 ** *p* < .01 | | | | | | | |

| Table S4. | |  |  |  |  |  |  |  |
| --- | --- | --- | --- | --- | --- | --- | --- | --- |
| *Whole-brain analysis for the interaction, main effect of valence, and main effect of instruction.* | | | | | | | | |
|  | | |  |  |  | MNI coordinates | | |
| Brain regions | | | Side | k | t | *x* | *y* | *z* |
|  | | |  |  |  |  |  |  |
| Interaction: Hypoactivation | | |  |  |  |  |  |  |
|  | Medial frontal gyrus | | R | 144 | 4.92 | 6 | 48 | 42 |
| Neutral > Positive | | |  |  |  |  |  |  |
|  | Fusiform gyrus | | L | 6489 | 18.67 | -27 | -54 | -9 |
|  | Inferior frontal gyrus, pars triangularis | | L | 595 | 8.13 | -54 | 24 | 21 |
|  | Inferior frontal gyrus, pars triangularis | | R | 431 | 8.02 | 54 | 27 | 27 |
|  | Supplementary motor area | | L | 227 | 5.39 | -12 | 18 | 66 |
|  | Middle frontal gyrus | | R | 307 | 5.25 | 24 | 15 | 48 |
| Watch > Up-regulate | | |  |  |  |  |  |  |
|  | Inferior parietal lobe | | R | 399 | 5.8 | 45 | -51 | 54 |
|  | Middle frontal gyrus | | R | 247 | 5.52 | 27 | 15 | 54 |
| *Note.* Clusters labeled according to the anatomical labeling (AAL) atlas (Tzourio-Mazoyer et al., 2002). Threshold: *p* < .001 (uncorrected) at the voxel level, and *p* < .05 with family-wise error (FWE) correction at the cluster level. | | | | | | | | |

| Table S5. | |  |  |
| --- | --- | --- | --- |
| *Neurosynth terms that show an association with activation in brain regions that are parametrically modulated by changes in self-reported affective valence during the fMRI task (*AffVal_fMRI_) | | | |
| Neurosynth term | | | *r* |
| Increased activation | | |  |
|  | amygdala | | .19 |
|  | fa | | .15 |
|  | hippocampus | | .15 |
|  | vmpfc | | .15 |
|  | amygdala hippocampus | | .14 |
|  | neutral | | .14 |
|  | arousal | | .14 |
|  | faces | | .14 |
|  | ventromedial | | .13 |
|  | ventromedial prefrontal | | .13 |
|  | callosum | | .13 |
|  | corpus callosum | | .13 |
|  | corpus | | .13 |
|  | cortex vmpfc | | .14 |
|  | amygdala response | | .14 |
|  | emotion | | .12 |
|  | putamen | | .12 |
|  | emotional | | .12 |
|  | hippocampal | | .12 |
|  | valence | | .12 |
|  | [cingulate cortex](http://neurosynth.org/analyses/terms/cingulate%20cortex) | | .14 |
|  | [fearful](http://neurosynth.org/analyses/terms/fearful) | | .14 |
|  | [pain](http://neurosynth.org/analyses/terms/pain) | | .14 |
|  | [limbic](http://neurosynth.org/analyses/terms/limbic) | | .14 |
|  | [reactivity](http://neurosynth.org/analyses/terms/reactivity) | | .14 |
| Decreased activation | | | |
|  | [frontal](http://neurosynth.org/analyses/terms/frontal) | | .26 |
|  | [working memory](http://neurosynth.org/analyses/terms/working%20memory) | | .22 |
|  | [working](http://neurosynth.org/analyses/terms/working) | | .22 |
|  | [inferior frontal](http://neurosynth.org/analyses/terms/inferior%20frontal) | | .21 |
|  | [task](http://neurosynth.org/analyses/terms/task) | | .21 |
|  | [parietal](http://neurosynth.org/analyses/terms/parietal) | | .20 |
|  | [inferior](http://neurosynth.org/analyses/terms/inferior) | | .20 |
|  | [comprehension](http://neurosynth.org/analyses/terms/comprehension) | | .19 |
|  | [sentences](http://neurosynth.org/analyses/terms/sentences) | | .18 |
|  | [sentence](http://neurosynth.org/analyses/terms/sentence) | | .17 |
|  | [demands](http://neurosynth.org/analyses/terms/demands) | | .17 |
|  | [linguistic](http://neurosynth.org/analyses/terms/linguistic) | | .17 |
|  | [tasks](http://neurosynth.org/analyses/terms/tasks) | | .16 |
|  | [dorsolateral](http://neurosynth.org/analyses/terms/dorsolateral) | | .16 |
|  | [language](http://neurosynth.org/analyses/terms/language) | | .16 |
|  | [parietal cortex](http://neurosynth.org/analyses/terms/parietal%20cortex) | | .16 |
|  | [frontoparietal](http://neurosynth.org/analyses/terms/frontoparietal) | | .16 |
|  | [fronto parietal](http://neurosynth.org/analyses/terms/fronto%20parietal) | | .16 |
|  | [prefrontal](http://neurosynth.org/analyses/terms/prefrontal) | | .15 |
|  | [frontal gyrus](http://neurosynth.org/analyses/terms/frontal%20gyrus) | | .15 |
|  | [theory mind](http://neurosynth.org/analyses/terms/theory%20mind) | | .15 |
|  | [syntactic](http://neurosynth.org/analyses/terms/syntactic) | | .14 |
|  | [mind tom](http://neurosynth.org/analyses/terms/mind%20tom) | | .14 |
|  | [speaker](http://neurosynth.org/analyses/terms/speaker) | | .14 |
|  | [semantic](http://neurosynth.org/analyses/terms/semantic) | | .13 |
